# *Mycoplasma pneumoniae* Community Acquired Respiratory Distress Syndrome toxin expression reveals growth phase and infection-dependent regulation

**DOI:** 10.1111/j.1365-2958.2010.07092.x

**Published:** 2010-03-03

**Authors:** T R Kannan, Oxana Musatovova, Sowmya Balasubramanian, Marianna Cagle, Jarrat L Jordan, Thomas M Krunkosky, Alan Davis, Robert D Hardy, Joel B Baseman

**Affiliations:** 1 Department of Microbiology and Immunology, The University of Texas Health Science Center at San AntonioSan Antonio, TX 78229, USA; 2 Centocor Research & Development, Inc.Radnor, PA 19087, USA; 3 Department of Anatomy and Radiology, University of GeorgiaAthens, GA 30602, USA; 4 Inverness Medical DiagnosticsScarborough, ME 04074, USA; 5 Department of Medicine, The University of Texas SouthwesternDallas, TX 75390, USA

## Abstract

*Mycoplasma pneumoniae* causes acute and chronic respiratory infections, including tracheobronchitis and community acquired pneumonia, and is linked to asthma and an array of extra-pulmonary disorders. Recently, we identified an ADP-ribosylating and vacuolating toxin of *M. pneumoniae*, designated Community Acquired Respiratory Distress Syndrome (CARDS) toxin. In this study we analysed CARDS toxin gene (annotated *mpn372*) transcription and identified its promoter. We also compared CARDS toxin mRNA and protein profiles in *M. pneumoniae* during distinct *in vitro* growth phases. CARDS toxin mRNA expression was maximal, but at low levels, during early exponential growth and declined sharply during mid-to-late log growth phases, which was in direct contrast to other mycoplasma genes examined. Between 7% and 10% of CARDS toxin was localized to the mycoplasma membrane at mid-exponential growth, which was reinforced by immunogold electron microscopy. No CARDS toxin was released into the medium. Upon *M. pneumoniae* infection of mammalian cells, increased expression of CARDS toxin mRNA was observed when compared with SP-4 broth-grown cultures. Further, confocal immunofluorescence microscopy revealed that *M. pneumoniae* readily expressed CARDS toxin during infection of differentiated normal human bronchial epithelial cells. Analysis of *M. pneumoniae*-infected mouse lung tissue revealed high expression of CARDS toxin per mycoplasma cell when compared with *M. pneumoniae* cells grown in SP-4 medium alone. Taken together, these studies indicate that CARDS toxin expression is carefully controlled by environmental cues that influence its transcription and translation. Further, the acceleration of CARDS toxin synthesis and accumulation *in vivo* is consistent with its role as a bona fide virulence determinant.

## Introduction

*Mycoplasma pneumoniae* is a significant bacterial pathogen of the airways and accounts for 20–30% of all community acquired pneumonia. It is also implicated in other airway diseases including asthma, and in extra-pulmonary manifestations, including neurological, gastrointestinal and dermatological disorders (Baseman and Tully, 1997; Waites and Talkington, 2004). *M. pneumoniae* colonizes the surfaces of epithelial cells and is also capable of invading host cells and establishing intracellular residence (Baseman *et al.*, 1995; Dallo and Baseman, 2000). Recently, we identified a unique virulence factor designated Community Acquired Respiratory Distress Syndrome (CARDS) toxin, an ADP-ribosylating and vacuolating toxin that binds alveolar surfactant protein A (Kannan *et al.*, 2005; Kannan and Baseman, 2006), likely contributing to additional colonization and pathogenic pathways. CARDS toxin ADP-ribosylates both similar and distinct human cell proteins when compared with the S1 catalytic subunit of pertussis toxin, leading to a cascade of events such as tissue disorganization, inflammation and airway dysfunction along with cell vacuolization (Kannan and Baseman, 2006). Hamster, murine and chimpanzee animal models and *in vitro* studies with tracheal organ cultures and human cell cultures have provided important insights in defining *M. pneumoniae* virulence potential (Hara *et al.*, 1974; Hu *et al.*, 1975; Barile *et al.*, 1981; Baseman *et al.*, 1995; Dallo and Baseman, 2000; Hardy *et al.*, 2002; Kannan and Baseman, 2006). Recently, normal human bronchial epithelial (NHBE) cells were used as a model to study *M. pneumoniae* interactions (Krunkosky *et al.*, 2007). NHBE cells maintained in the air–liquid interface culture system exhibit well differentiated heterogenous populations of ciliated and secretory cells remarkably similar to the lumen of the airway.

Clearly, *M. pneumoniae* must co-ordinate a wide range of virulence factors and circumvent host defenses in order to colonize, propagate, internalize, persist and be transmitted. Transcriptional and translational regulation in *M. pneumoniae* appears to be unique compared with other procaryotes, as this mycoplasma possesses only one authentic sigma factor and a limited number of genes encoding typical transcriptional and translational regulatory elements (Himmelreich *et al.*, 1996; Dandekar *et al.*, 2000), including helix–turn–helix (HTH) motifs (Samuelsson *et al.*, 1997). Although there is no classical two-component system in *M. pneumoniae* (Himmelreich *et al.*, 1996), there is evidence that *M. pneumoniae* is able to differentially regulate gene expression in response to environmental stimuli. For example, transcriptional regulation of mycoplasma heat shock genes has been observed in *M. pneumoniae* and other pathogenic *Mycoplasma* species (Weiner *et al.*, 2003; Madsen *et al.*, 2006; Musatovova *et al.*, 2006; Chang *et al.*, 2008; Kannan *et al.*, 2008). Differential expression of lipoprotein genes in *M. pneumoniae* after acidic and oxidative stresses (Hallamaa *et al.*, 2008) and regulation of *ackA* (acetate kinase) and *ldh* (lactate dehydrogenase) genes by glycerol (Halbedel *et al.*, 2007) were recently reported. After contact with human lung epithelial cells, distinct patterns of *M. pneumoniae* lipoprotein gene expression were observed (Hallamaa *et al.*, 2008). While upregulation of four heat shock genes (*dnaJ*, *dnaK*, *lon* and *clpB*) can be attributed to a functioning HrcA-CIRCE regulatory apparatus (Narberhaus, 1999; Weiner *et al.*, 2003), in most cases the mechanisms of gene expression and regulation have not been identified. In addition, little is known regarding post-transcriptional and translational controls in *M. pneumoniae*, although evidence from two-dimensional gel electrophoresis, lipoprotein characterization, cleavage of signal peptides and phosphorylation analyses suggest that these processes exist (Razin *et al.*, 1998; Regula *et al.*, 2000; Ueberle *et al.*, 2002; Jaffe *et al.*, 2004).

Because only a paucity of data is available concerning regulation of virulence genes in *M. pneumoniae*, we focused on the identification of the putative promoter of the *cards* gene and monitored *cards* transcript levels during *M. pneumoniae in vitro* growth and after contact with host cells. We further demonstrated surface localization of CARDS toxin on intact mycoplasma cells with no evidence for release into the environment. Interestingly, we noted substantial increases in the synthesis of CARDS toxin protein per mycoplasma cell in infected mice. These data suggest that understanding how airway-associated environmental signals regulate CARDS toxin expression should provide important clues concerning *M. pneumoniae* virulence and associated pathologies.

## Results

### *cards* gene organization and promoter mapping

**Fig. 1:**
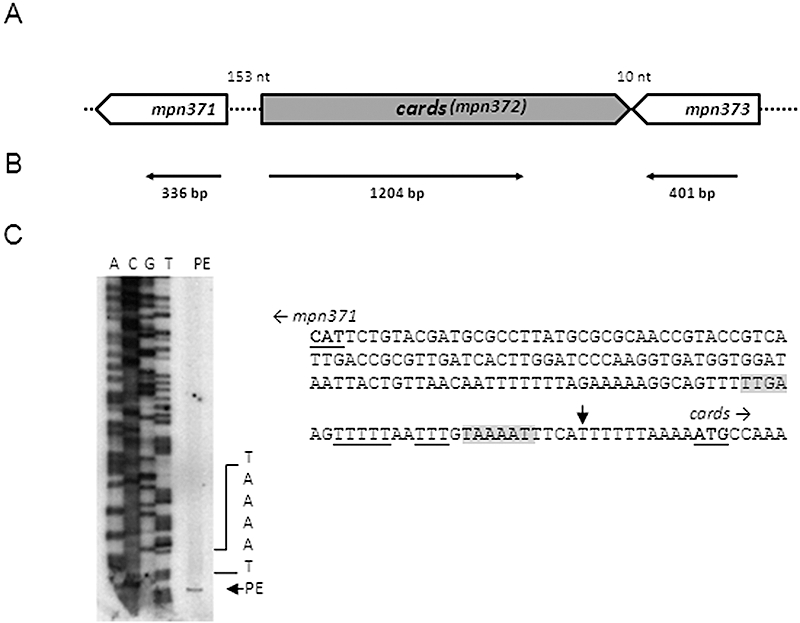
Chromosomal organization and transcriptional and primer extension analyses of *cards* (*mpn372*). A. Schematic of *cards* and its surrounding genes. *mpn371* and *mpn373* encode hypothetical proteins. The orientation of individual genes is indicated (open arrows), and numbers represent the length of intergenic regions. B. Transcriptional analysis. RT-PCR was performed on total RNA isolated from *M. pneumoniae* cells. The thin horizontal arrows represent regions that were amplified to confirm transcription of individual genes, and numbers represent sizes of RT-PCR amplified products. C. Primer extension (PE) analysis and characterization of *cards* gene promoter. The PE product was separated by electrophoresis and analysed alongside DNA sequence (A, T, C and G). The sequence of the intergenic region separating *mpn371* and *cards* is provided. The translational start sites of *mpn371* and *cards* are indicated (bold letters). A vertical arrow designates the transcriptional start point identified for *cards*. Putative Pribnow box (−10 site), along with *M. pneumoniae* conserved TTGA sequences, are shaded in gray colour. Poly-T tracts are underlined.

**Fig. 2:**
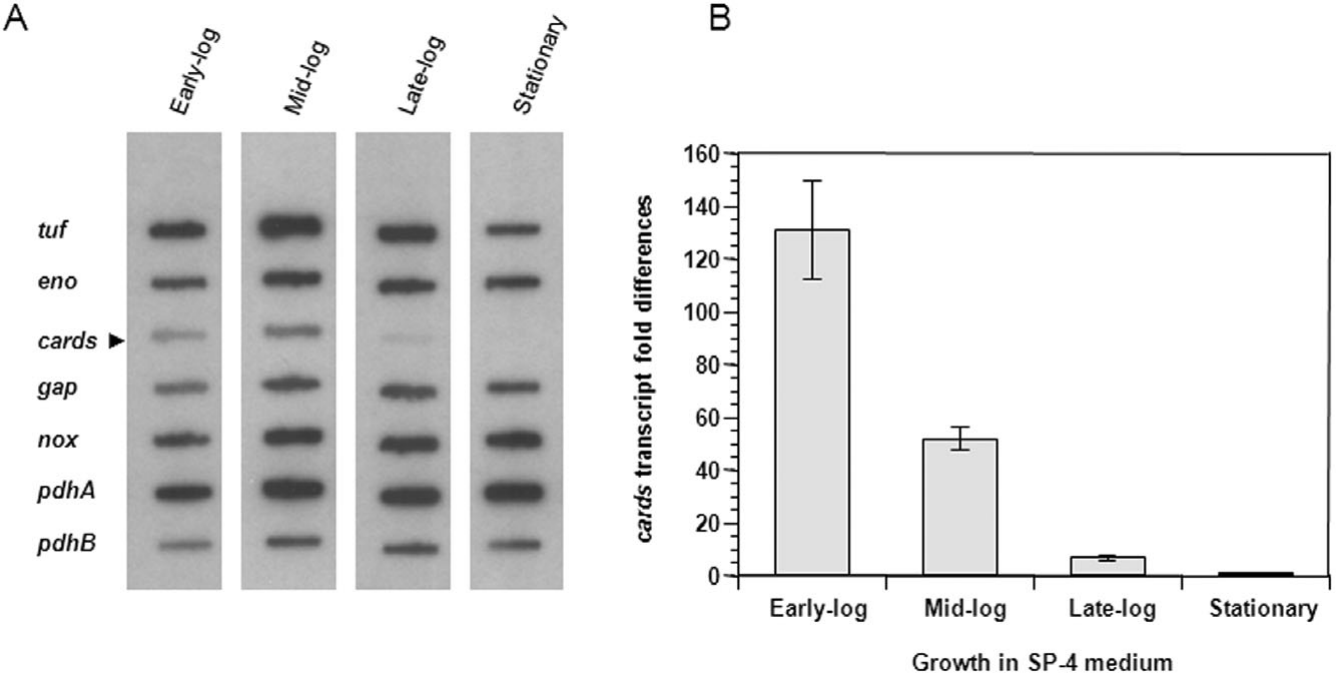
Transcription of *cards* during *in vitro* growth of *M. pneumoniae* in SP-4 broth. A. Expression of *cards* along with other genes was analysed by DNA slot blot. *M. pneumoniae* gene-specific PCR products (Table S2) were blotted onto Zeta probe membranes. *M. pneumoniae* S1 cells were grown and harvested at early-log (24 h), mid-log (48 h), late-log (72 h) and stationary phases (120 h). ^32^P-labelled cDNAs generated by reverse transcription of isolated total RNA were used as hybridization probes. Experiments were repeated three times. B. Expression of *cards* by relative real-time quantitative RT-PCR (qRT-PCR). *M. pneumoniae* S1 cells were grown and RNA isolated at specific growth phases. Real-time qRT-PCR was performed using SYBR green chemistry as detailed in *Experimental procedures*. Experiments were repeated two times. The average fold differences in expression levels of cards mRNA at various time points (when compared with the stationary phase) and standard deviations (SD) are presented.

In *M. pneumoniae* reference strain M129, the *cards* gene (*mpn372*, nucleotides 444341-446116) is flanked by *mpn371* (nucleotides 444187-443552) and *mpn373* (nucleotides 446741-446127) genes. Both *mpn371* and *mpn373* genes are transcribed from the complementary strand, in contrast to *cards*, and encode hypothetical proteins of unknown functions (Fig. 1A). *cards* is separated from upstream *mpn371* by a 153-nucleotide long intergenic region (head-to-head orientation) and from downstream *mpn373* by a 10-nucleotide short intergenic region (tail-to-tail orientation). Analysis of all three genes by reverse transcription PCR (RT-PCR) revealed three transcripts of expected polarity (Fig. 1B). Based on this gene organization, we predicted *cards* to have its own promoter. Primer extension (PE) analysis revealed a single transcriptional start point (TSP) at 10 nucleotides upstream of the *cards* translational start (Fig. 1C). Further examination of the sequence upstream of the TSP revealed additional consensus features of *M. pneumoniae* promoters, such as the presence of a −10 element (Pribnow box; TAAAAT; Fig. 1C) four nucleotides upstream from the identified TSP. The sequence immediately 5′ to the −10 element was AT-rich and contained polythymidine tracts (3 and 5 residues; Fig. 1C). While there was no strong consensus in the −35 region, the relatively conserved *M. pneumoniae* promoter-unique TTGA (Weiner *et al.*, 2000) was found upstream of the −10 region. PE analyses did not identify individual transcriptional starts for *mpn371* or *mpn373*. Although transcription of *cards* was readily confirmed by RT-PCR and low amounts of transcript were repeatedly demonstrated by slot blot analysis (Fig. 2A), Northern blot analysis did not detect *cards* transcripts (data not shown) possibly because of low-level expression.

### Is expression of *cards* temporal?

To determine the dynamics of *cards* transcript expression during *in vitro* mycoplasma growth in SP-4 broth, we performed slot blot analyses with total RNA from early-, mid-, late-exponential and stationary growth phases of *M. pneumoniae* cells. *cards* mRNA levels were compared with differential mRNA expression of selected *M. pneumoniae* metabolic genes (*tuf*, *pdhB*, *pdhA*, *gap*, *eno* and *nox*; Fig. 2A) and adherence- and heat shock-related genes (Fig. S1). Results indicated that *cards* expression occurred at low levels during early stages of growth, which decreased dramatically as growth progressed (Fig. 2A). In contrast, other genes exhibited relatively stable or increased gene transcription throughout growth (*eno*, *gap*, *nox*, *pdhA* and *pdhB*) while *tuf* exhibited a similar pattern except at stationary phase where a noticeable decline in transcription occurred (Fig. 2A). Adherence- and heat shock-related genes exhibited relatively stable or diminished transcription profiles during mid-to-late log growth phases but, in all cases, increased transcription was observed during later stages of growth in direct contrast to *cards* profiles (Fig. S1). To further confirm the specificity of *cards* expression during different mycoplasma growth intervals, we performed real-time quantitative reverse transcriptase polymerase chain reaction (qRT-PCR). As indicated in Fig. 2B, the expression of CARDS toxin transcription decreased dramatically (∼130-fold) between early-log and stationary phases.

### Total *M. pneumoniae* cell protein profiles at different growth stages

**Fig. 3:**
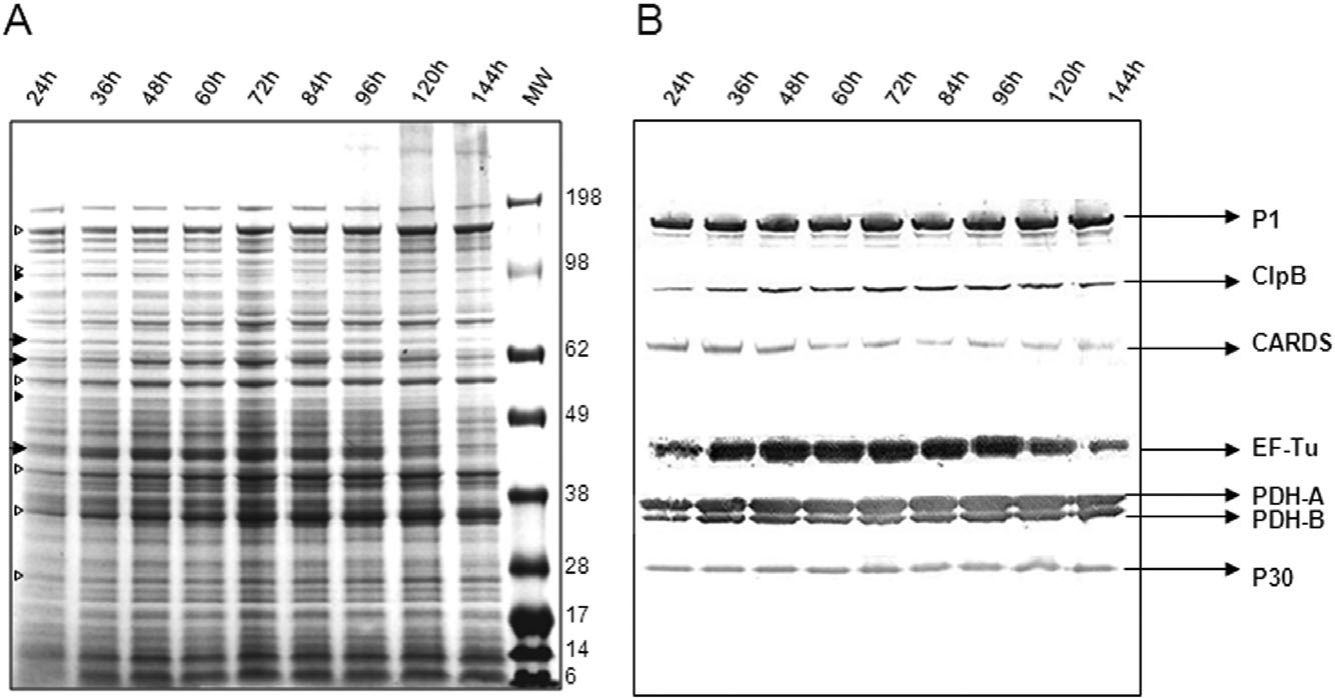
Differential synthesis of *M. pneumoniae* proteins during *in vitro* growth. *M. pneumoniae* S1 cells were grown in SP-4 broth for various times and samples collected and processed as described in *Experimental procedures*. A. Equal amounts of S1 total cell proteins resolved on 4–12% Nu-PAGE gradient gel and stained with Coomassie Blue. Open arrows: increased protein intensity during early-to-late growth stages. Small closed arrows: decreased protein intensity during mid-to-late growth stages. Large closed arrows: increased protein intensity during stationary stage. B. Parallel gel transferred to nitrocellulose membrane for immunoblotting. Membrane strips were cut and treated with respective *M. pneumoniae* antibodies. Experiments were repeated two times.

**Fig. 4:**
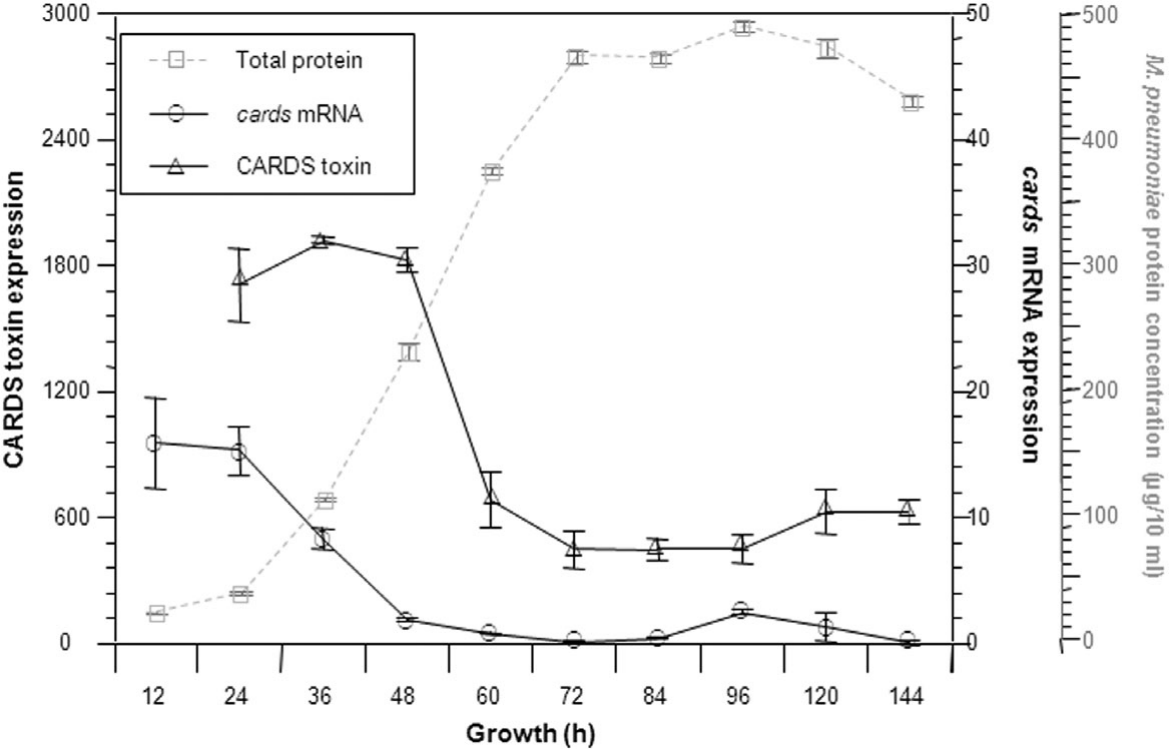
Growth phase-dependent variations in *cards* mRNA and CARDS toxin protein levels in *M. pneumoniae* broth cultures. *M. pneumoniae* cells were grown at 37°C for 12, 24, 36, 48, 60, 72, 84, 96 and 120 h and harvested at each time interval for analysis. Experiments were repeated two times. Levels of *cards* transcript are estimated by DNA slot blot (average value ± SD), CARDS toxin protein levels are quantified by immunoblot, and mycoplasma growth (total protein) is indicated in grey dotted lines (average value ± SD), and expression levels are presented in arbitrary values.

In order to compare CARDS toxin protein levels with other mycoplasma proteins, we harvested mycoplasma cells during an extended (12–144 h) growth period in SP-4 broth and analysed protein profiles (Fig. 3A). Four distinct protein patterns were readily observed based upon SDS-polyacrylamide gel electrophoresis (PAGE) profiles and image analysis. One protein category exhibited increased intensity as mycoplasma growth proceeded from early to late stages (open arrow). Another category showed early intensity, which subsided or disappeared during mid-to-late log phase (closed small arrow). The third category exhibited increased intensity throughout logarithmic growth, which decreased during the stationary stage (large closed arrow). The last category represented proteins that remained mostly unchanged (no arrow). We selected specific proteins representing adherence- and stress-related functions (adhesin P1, adhesin P30, PDH-A, PDH-B, EF-Tu and ClpB) for comparison with CARDS toxin using immunoblotting (Fig. 3B). The intensities of immunoreactive bands demonstrated that CARDS toxin protein levels peaked between 24 and 48 h and declined to the lowest levels at 60 h, which continued over the remaining growth period (60–96 h; Fig. 3B). Other proteins demonstrated levels of detection consistent with their pattern of mRNA transcripts (Fig. 2). Growth stage-related dynamics of CARDS toxin mRNA and protein expression are depicted in Fig. 4 and are compared with the progression of culture growth (early-log through stationary phases). These quantitative data clearly reinforce the selective early synthesis of *cards* transcripts and CARDS toxin protein (Fig. 4). Note the relative stability of CARDS toxin following *cards* transcriptional decline (i.e. 60–144 h).

### CARDS toxin location in mycoplasma membranes

**Fig. 5:**
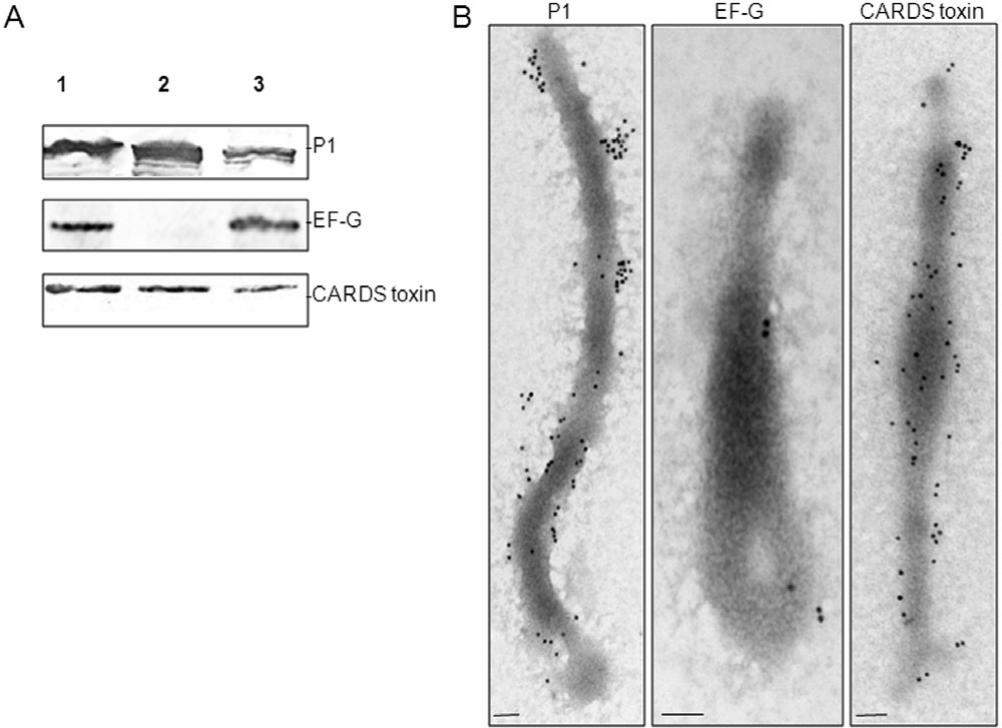
Cell fractionation of *M. pneumoniae* and localization of CARDS toxin. A. Total lysate, membrane and cytoplasmic fractions of *M. pneumoniae* cells were isolated as described in *Experimental procedures*. Equal amounts of mycoplasma proteins from each fraction were resolved on 4–12% gradient Nu-PAGE gels and transferred to nitrocellulose membranes. Specific proteins were identified by immunoblotting for the presence of CARDS toxin, cytoplasmic EF-G and membrane adhesin P1. Lane 1. total cell lysate; lane 2. membrane fraction; and lane 3. cytoplasmic fraction. B. Immunogold electron microscopy detection of CARDS toxin and control proteins P1 and EF-G on intact *M. pneumoniae* cells. *M. pneumoniae* cells were gold particle-labelled with anti-P1, anti-EF-G and anti-CARDS toxin antibodies as described in *Experimental procedures*. Mycoplasma cells were visualized with a JEOL 1230 transmission electron microscope. Gold labelling of CARDS toxin revealed surface membrane distribution throughout the *M. pneumoniae* membrane and tip organelle (bar = 0.1 µm). Experiments were repeated two times.

To assess the location of CARDS toxin in *M. pneumoniae* cells, we performed immunoblot analyses on cellular fractions of *M. pneumoniae* clinical isolate S1. Whole cell lysates and cytoplasmic, membrane and culture supernatant preparations were quantified for the presence of CARDS toxin as described in *Experimental procedures*. In parallel, the localization of P1 (major surface-associated adhesin protein) and EF-G (intracellular cytosolic protein) was used as control. As shown in Fig. 5A, at 48 h of mycoplasma growth, CARDS toxin was detected in the density gradient-purified membrane fraction, which is free of intracellular protein contamination (note the absence of EF-G in lane 2). When compared with total CARDS toxin concentrations, 7–10% of CARDS toxin was associated with the mycoplasma membrane fraction. No full-length CARDS toxin was detected in culture supernatant (data not shown). Occasionally, we observed two minor CARDS toxin-related immunoreactive bands of low molecular weight in the supernatant of 72 and 96 h cultures, which may represent degraded or processed CARDS toxin (data not shown). Immunogold labelling and electron microscopic analysis revealed CARDS toxin distribution over the entire surface of *M. pneumoniae* cells, including the tip organelle, a pattern similar to the major adhesin protein P1 (Fig. 5B).

### Expression of CARDS toxin in *M. pneumoniae* during co-incubation with HeLa and differentiated NHBE cells

**Fig. 6:**
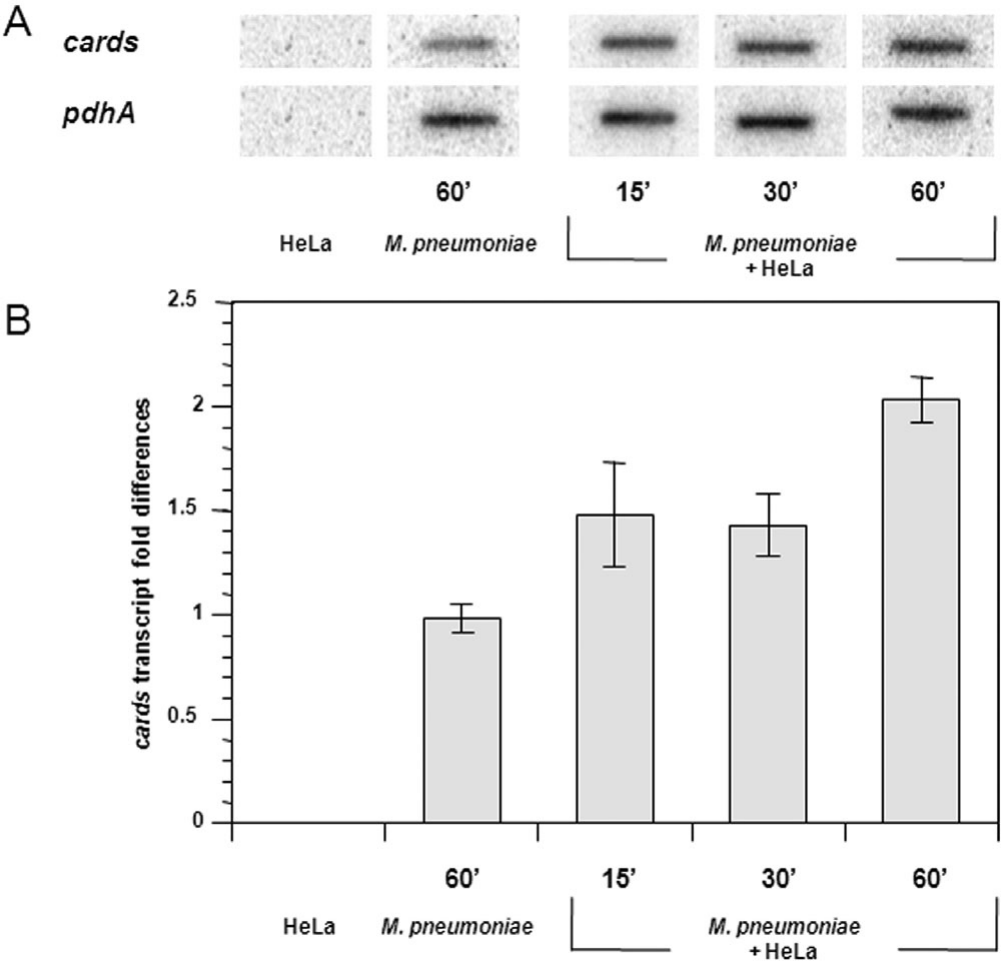
Differential expression of *cards* mRNA in *M. pneumoniae* during co-incubation with HeLa cells. A. Autoradiographic DNA slot blot analysis for *cards* and *pdhA* genes. *M. pneumoniae*-specific primers were used to generate ^32^P-labelled cDNAs from total RNA isolated from HeLa cells alone, *M. pneumoniae* cells alone or *M. pneumoniae* cells co-incubated with HeLa cells for different time intervals. Experiments were repeated three times. B. Relative quantification of *cards* mRNA by real-time qRT-PCR. *M. pneumoniae* S1 cells were co-incubated with HeLa cells for 15, 30 and 60 min as described in *Experimental procedures*, and RNA was isolated. Real-time qRT-PCR was performed using SYBR green chemistry and *pdhA* as a normalizer. Experiments were repeated two times. The average fold differences in expression levels of *cards* mRNA at various time points (when compared with the *cards* mRNA level at time 0) and SD are presented.

**Fig. 7:**
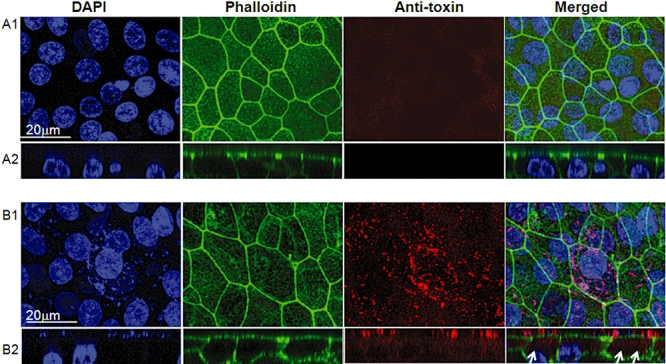
Localization of CARDS toxin in *M. pneumoniae*-infected NHBE cells. Fully differentiated NHBE cells grown in air–liquid interface were infected with *M. pneumoniae*. Thirty-eight hours post infection, cultures were washed to remove unbound mycoplasmas, submerged in fixative, processed for laser scanning confocal microscopy and screened using mouse polyclonal anti-CARDS toxin sera. Luminal (A1 and B1) and cross-sectional (A2 and B2) views of uninfected (A1 and A2) and infected (B1 and B2) NHBE cells are presented. In the latter case, surface colonization by mycoplasmas and synthesis and distribution of CARDS toxin during infection are readily observed (red). Cross-sectional views of infected cells reveal intracellular localization of CARDS toxin (arrows). Also, DAPI staining indicates the abundance of surface associated *M. pneumoniae* DNA (small blue dots on the respiratory cell surface; B1 and B2). Merged images of the cross-sectional view indicate colocalization of CARDS toxin with *M. pneumoniae* at respiratory cell surfaces and also demonstrate that toxin is detectable inside target cells, possibly free of associated *M. pneumoniae*. Experiments were repeated three times.

To evaluate the potential role of host cell factors in *cards* expression during infection, we incubated HeLa cells with *M. pneumoniae* and monitored the expression of *cards* after 15, 30 and 60 min. As appears in slot blot (Fig. 6A), higher expression of *cards* mRNA was readily detectable when *M. pneumoniae* cells were co-incubated with HeLa cells in contrast to growth in broth culture alone. Real-time qRT-PCR data revealed that constant levels of *cards* transcription were maintained during the 60 min time frame in SP-4 broth whereas a fold increase in *cards* transcript levels was detected in *M. pneumoniae* cells after 1 h co-incubation with HeLa cells. To further reinforce the effect of co-incubation on CARDS toxin expression in *M. pneumoniae* cells, we used confocal immunofluorescence microscopy to detect CARDS toxin synthesis during mycoplasma infection of differentiated NHBE cells. Mycoplasmas were distributed over NHBE cell surfaces with concomitant CARDS toxin synthesis. *Z*-section analysis of infected NHBE cells revealed CARDS toxin both outside and inside NHBE cells (Fig. 7).

### Expression of CARDS toxin during infection of mice

**Fig. 8:**
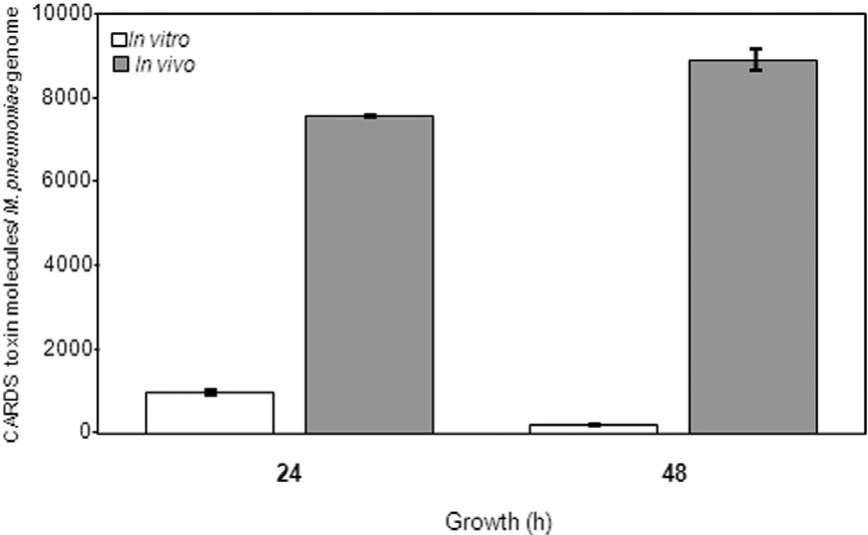
Differential expression of CARDS toxin in mice. Mice were infected intranasally with *M. pneumoniae*, and lungs were harvested after 24 and 48 h of infection and analysed for the presence of CARDS toxin molecules per *M. pneumoniae* cell by antigen capture assay and quantitative real-time PCR (qPCR). In parallel, *M. pneumoniae* cells were grown in SP-4 broth, harvested and analysed similarly. Numbers of CARDS toxin molecules per mycoplasma genome in SP-4-grown *M. pneumoniae* cells (*in vitro*) or in lungs of infected mice (*in vivo*) are represented by bar diagrams. Experiments were repeated two times with three to five animals of each group and at each time point.

In *M. pneumoniae*-infected pneumonia patients, evidence for seroconversion against CARDS toxin is striking (Kannan and Baseman, 2006) indicating that CARDS toxin is expressed during human infection and is highly immunogenic. This contrasts with the low transient expression observed during *M. pneumoniae* growth in SP-4 broth (Figs 2 and 3). Since we observed increased *cards* transcript levels and readily measurable CARDS toxin production following co-incubation of mycoplasmas with host cells (Figs 6 and 7), we analysed the expression of CARDS toxin during *in vivo* infection. In intranasally inoculated mice, *M. pneumoniae* expressed CARDS toxin per mycoplasma genome in lung tissues at much higher levels (∼8–40-fold at 24–48 h post infection, respectively) than during growth in SP-4 broth (Fig. 8).

## Discussion

In this report we describe growth-regulated gene expression in *M. pneumoniae*, with special interest in the dynamics of *cards* mRNA expression and CARDS toxin protein synthesis *in vitro* and *in vivo*. Growth conditions are invariably different in the test tube versus those encountered during the course of infection. Only recently have transcriptional studies been performed to examine *M. pneumoniae* responses to environmental cues (Weiner *et al.*, 2003; Halbedel *et al.*, 2004; 2007; Kannan *et al.*, 2008). None have focused on monitoring bona fide virulence determinants, like CARDS toxin. Therefore, the information provided here represents an essential step in monitoring how *M. pneumoniae* may behave during the infectious process. With the availability of *M. pneumoniae*-purified recombinant proteins and specific antibody reagents, we were able to quantify and correlate transcriptional analyses with translational responses between CARDS toxin and a range of *M. pneumoniae* proteins.

Using *M. pneumoniae* clinical strain S1 (Kannan and Baseman, 2006; Musatovova *et al.*, 2008) we demonstrated transcription of all three genes (*cards* and its adjacent genes: *mpn371*-*mpn373*) in their predicted orientations (Fig. 1). Previously, corresponding gene transcripts were analysed in *M. pneumoniae* strain M129 before and after temperature shift (Weiner *et al.*, 2003). We also sequenced the corresponding regions in strain S1 and found very minimal differences in the sequences (see Fig. S2). As shown in Fig. 1C, the *cards* transcript contains a short 5′-untranslated region (transcription start point at T^−10^). Because *cards* is followed by a gene with opposite polarity (Fig. 1A), we expected *cards* to be ∼1.8 kb single gene transcript. Recently, such monocistronic messages were shown for *M. pneumoniae* genes *ldh* and *ptsH* (Halbedel *et al.*, 2004; 2007;). However, repeated Northern blots did not permit visualization of any clear band for *cards*. It is possible that the low amount of transcript we detected (Figs 2 and 4) is further degraded during isolation. Also possible and contrary to *ldh* and *ptsH* genes, a transcription termination signal is not predicted for *cards* (Ermolaeva *et al.*, 2000), and transcripts of different lengths may be generated.

We investigated CARDS toxin synthesis by measuring changes in *cards* transcript levels and comparing these data with CARDS toxin protein concentrations. This is important because the relationship between mRNA levels and rates of protein synthesis can be non-linear (Vanbogelen *et al.*, 1999). Moreover, protein analysis makes it possible to study critical post-translational control mechanisms, such as protein modification and stability, which may contribute greatly to the ultimate properties of a given protein. Although we observed a surprising demise of *cards* transcripts from early-to-mid log mycoplasma growth in SP-4 medium (Fig. 2B), we still detected CARDS toxin protein levels late into stationary phase (Figs 3 and 4). The detection of CARDS toxin many hours after the end of exponential growth can be explained either by toxin stability and/or by its continued low-level synthesis as a result of residual *de novo* transcription. In contrast to CARDS toxin, the toxins of *Bortedella pertussis* (Rambow-Larsen and Weiss, 2004) and *Staphylococcus aureus* (Payne, 2003) are maximally expressed at mid-to-late exponential growth phases (pertussis toxin) and late- to post-exponential growth phases (α toxin). Similar high-level expression of toxins during late-log and stationary phase growth-related time points was observed in other bacteria (Thompson *et al.*, 2003; Wagner *et al.*, 2003; Snyder *et al.*, 2004). Distinct from *cards* gene transcription, we observed high-level expression of adherence- and stress-related genes during late-log and stationary stage growth of *M. pneumoniae*, and similar growth dependent expression patterns of adherence- and stress-related genes have been observed in other bacteria (Strohmaier *et al.*, 1995; Xu and Johnson, 1995; Nicholson *et al.*, 2003; Thompson *et al.*, 2003).

Earlier, we reported that CARDS toxin was a surface-associated, trypsin-sensitive surfactant protein A binding 68 kDa protein (Kannan *et al.*, 2005). In the current study, we further reinforced its cell-associated distribution and lack of secretion into the medium during mycoplasma growth in SP-4 broth. Interestingly, only 7–10% of the toxin appears membrane-bound (Fig. 5A), and immunoelectron microscopy (Fig. 5B) further confirmed CARDS toxin surface localization. The majority of toxin is detected in the cytoplasm, which was confirmed by immunoblot comparisons between concentrated supernatant (ammonium sulphate precipitated and desalted), total mycoplasma lysate, membrane fractions and cytoplasmic fractions (Fig. 5A). Consistent with these results, we could not identify signal sequence specific for secreted proteins in CARDS toxin. Because higher expression of CARDS toxin is observed when mycoplasmas are co-incubated with host cells, it is possible that redistribution of toxin from the mycoplasma cytoplasm to mycoplasma membrane surface occurs, facilitating toxin-mediated events. For example, although most classical bacterial protein toxins are secreted into the surroundings, some are displayed on the bacterial surface or released upon contact with the host. The latter could be a possible scenario for CARDS toxin, as toxin colocalizes with mycoplasmas during colonization of host cells and can be detected inside NHBE cells based upon the merging of DAPI staining with anti-toxin immunostaining (Fig. 7). Therefore, mycoplasmas and toxin together appear to follow predictable patterns of other toxin-producing bacteria by evading host defenses, adhering to cells and tissue matrices, spreading within the host, eliciting inflammatory pathways and degrading host target cells and tissues for nutritional and survival benefits (Finlay and Falkow, 1997). Purified toxins of cholera, tetanus and diphtheria also serve critical functions in establishing infection and associated pathologies (Finlay and Falkow, 1997). This is consistent with the characteristic ciliostasis, cytoplasmic vacuolization, nuclear swelling, and extensive epithelial cell fragmentation and sloughing observed in hamster tracheal organ cultures infected with *M. pneumoniae* (Hu *et al.*, 1976; Murphy *et al.*, 1980). Importantly, this pathological pattern parallels observations when CARDS toxin alone is added to baboon tracheal organ cultures (Kannan and Baseman, 2006).

As with virulence factors in other pathogens, CARDS toxin probably relies on other mycoplasma components to fully potentiate its expression, delivery and impact on the host. Other possible microbial components include gene and protein regulatory systems, adherence factors for colonization and invasion and possible secretion systems for delivery of specific pathogenic products. Because CARDS toxin location is also associated with the tip organelle in addition to its membrane distribution (Fig. 5B), a likely scenario is that tip-mediated adherence brings *M. pneumoniae* into close contact with host target cells for delivery of toxin as observed with vibrio (Srivastava *et al.*, 1980). In addition, CARDS toxin also can bind to mammalian cell surfaces through SP-A-like molecules and facilitate colonization, similar to *B. pertussis* pertussis toxin that appears to assist bacterial adherence to mammalian cells (Tuomanen and Weiss, 1985).

While CARDS toxin expression is transient during *M. pneumoniae* growth in SP-4 medium, it was appreciably increased during contact with human cells (Figs 6 and 7) suggesting the influence of host cell factors. This was further supported by the many-fold increase in CARDS toxin protein molecules per *M. pneumoniae* cell ratio in mice versus broth culture (Fig. 8). The expression of bacterial toxins is generally tightly regulated and controlled at the transcriptional level by virtue of repression and/or transactivation via specific regulatory proteins (Gallegos *et al.*, 1997; Rood, 1998; Hirst, 1999; Payne, 2003; Stibitz, 2003). The transient and decreased expression of CARDS toxin in *M. pneumoniae* during *in vitro* growth, *cards* transcriptional upregulation during co-incubation with host cells, detection of CARDS toxin in infected NHBE cells, CARDS toxin increased synthesis per mycoplasma cell in mice and the high-titre seroconversion to CARDS toxin in infected patients (Kannan and Baseman, 2006) suggest that the production of CARDS toxin is also controlled *in vivo* by environmental host signals. Uncovering the physiological cues that influence *in vivo* mycoplasma growth and CARDS toxin expression will be critical in clarifying the pathogen–host interaction and in devising strategies, including new antimicrobial agents, targeted immunotherapies and effective vaccines, to control and prevent *M. pneumoniae*-mediated disease progression.

## Experimental procedures

### Bacterial strains, human tissue cultures, growth conditions and collection of samples

For growth curve studies, *M. pneumoniae* S1 cells (Musatovova *et al.*, 2008) were grown in 400 ml of SP-4 medium (4 × 100 ml in 150 cm^2^ tissue flasks) and incubated at 37°C for 72 h. Surface-attached mycoplasmas were harvested, concentrated, pooled and divided into forty 150 cm^2^ tissue culture flasks, which were incubated at 37°C for 12, 24, 36, 48, 60, 72, 84, 96 and 120 h. Three flasks were collected at each time interval [one for RNA isolation and two for protein characterization, colony counts, optical density (OD_600_) determinations and protein measurements]. For OD_600_ reading, cells were washed in phosphate-buffered saline (PBS, pH 7.4) and scraped and passed through syringes with 25-gauge needles. Additional flasks were used at appropriate time intervals for membrane isolation and other analyses described below. Each experiment was repeated at least three times. All samples were stored at −80°C until use. For evidence of secreted CARDS toxin during *M. pneumoniae* growth in SP-4 broth, culture-grown supernatant samples were filter-sterilized using 0.2 µm filters to remove mycoplasmas and then concentrated using ammonium sulphate (30–80% saturation). Supernatants were dialysed with PBS and analysed for the presence of CARDS toxin by immunoblot. Recombinant *Escherichia coli* cells were grown in Luria–Bertani (LB) broth or LB agar containing ampicillin (100 µg ml^−1^) at 37°C.

Normal human bronchial epithelial cells were grown in air–liquid interface as previously described (Krunkosky *et al.*, 2007). Briefly, NHBE cells (Cambrex, San Diego, CA) were seeded onto Transwell-clear culture inserts (24.5, 0.45 mm pore size; Costar, Cambridge, MA) thin-coated with rat tail collagen type I (Collaborative Res., Bedford, MA). The apical surfaces of NHBE cells were exposed to a humidified 95% air/5% CO_2_ environment and medium changed daily during 21–28 days of air–liquid culture. To study expression and localization of CARDS toxin during *M. pneumoniae* infection of NHBE cells, stocks of clinical strain S1 were quantified by dilution and plating. Prior to infection, *M. pneumoniae* inocula were passed 10× through 25-gauge needles and added apically to each culture well (50 mycoplasmas: 1 NHBE cell) for specific times. Total epithelial cells exposed to apical luminal surfaces in 1 cm^2^ culture inserts were estimated to be approximately 450 000 by examination of confocal microscopic images from multiple fields of fully differentiated NHBE cells. Each experiment was repeated in triplicate.

To further examine experimental conditions that could influence the degree of expression of *cards* transcripts in *M. pneumoniae* cells, we incubated mycoplasma cell monolayers alone or with HeLa cells (ATCC), the latter had been resuspended in SP-4 broth supernatant (0.2 µm filtered) from 48 h depleted mycoplasma growth medium in order to eliminate the effect of fresh SP-4 components on *cards* expression. Ten millilitres of depleted mycoplasma growth medium suspended HeLa cells was overlaid on mycoplasma monolayers for 15, 30 and 60 min. Test samples were collected at specific intervals, RNA isolated each time and DNA blot slot blots and real-time qRT-PCR performed. Experiments were repeated at least two times for each experiment.

### DNA manipulations, primer design and DNA slot blot construction

*Mycoplasma pneumoniae* chromosomal DNA was isolated using Easy-DNA kit (Invitrogen, Carlsbad, CA), quantified by OD_260_, and 100 ng were used in amplification reactions. Sequences of selected genes were obtained from NCBI databases, and primers for PE analysis, RT-PCR and amplification of genomic regions of selected genes were designed (Table S1).

To obtain sequencing templates in PE analysis, corresponding regions of the *M. pneumoniae* chromosome were amplified using specific primers and high-fidelity AccuTaq LA DNA polymerase (Sigma-Aldrich, St. Louis, MO). Generated amplicons were TA-cloned into pCR2.1 and transformed into TOP10 cells following manufacturer's instruction (Invitrogen). Recombinant plasmids were purified from *E. coli* cultures using the QIAprep spin kit (Qiagen, Valencia, CA).

For DNA slot blot construction, specific regions of selected *M. pneumoniae* genes were amplified using corresponding primers (Table S2) and high-fidelity AccuTaq LA DNA polymerase (Sigma-Aldrich). All generated amplicons were purified by gel extraction, and 200 ng of each specific PCR product was blotted in triplicate on Zeta probe membranes by using Bio-Dot SF microfiltration apparatus as suggested by the manufacturer (Bio-Rad Laboratories, Hercules, CA).

### Transcriptional analysis

Total RNA from *M. pneumoniae* was isolated with RNase-free reagents and plastic ware. Surface-adherent mycoplasmas alone and *M. pneumoniae* cells co-incubated with HeLa cells were washed twice with 25 ml of ice-cold PBS. Sixteen millilitres of TriReagent (Sigma-Aldrich) was added to each T150 flask, and RNA was extracted as suggested by the manufacturer. Pellets were dissolved in RNase-free water and quantified at 260 nm. RNA was subjected to DNase I treatment (Gibco-BRL) prior to further use.

To determine TSP we performed PE with primers that were end-labelled with [γ-^32^P]-ATP using polynucleotide kinase (United States Biochemical, Cleveland, OH). Twenty-five micrograms of total mycoplasma RNA and 15–20 ng of radiolabelled primer were heated to 70°C for 10 min and cooled slowly. After annealing, 5 µl of 10× reverse transcription buffer (200 mM Tris-HCl, pH 8.4, 500 mM KCl), 5 µl of 25 mM MgCl_2_, 5 µl of 100 mM dithiothreitol, 2.5 µl of 5 mM dNTPs and 1 µl of Superscript II RT (Gibco-BRL) were added, and PE was performed at 42°C. PE reactions were terminated by incubation at 70°C for 15 min, *E. coli* RNase H (2 units per sample) added, and incubation continued at 37°C for 20 min. Individual PE products were phenol–chloroform extracted, precipitated with ethanol and dissolved in 6 µl of H_2_O and 3 µl of loading buffer. Five microlitres of each sample was analysed in 6% sequencing gels alongside sequencing reactions (dsDNA Cycle Sequencing System, Gibco-BRL) generated with the same end-labelled oligonucleotide and recombinant plasmid containing corresponding sequence.

Transcription of *mpn371*, *cards* and *mpn373* genes was demonstrated by RT-PCR using isolated RNA. Five micrograms of total RNA was subjected to DNase I treatment (Gibco-BRL), and individual reactions were performed with corresponding antisense primers (Table S1) and SuperScript first-strand synthesis system as suggested by the manufacturer (Gibco-BRL). Generated complementary DNAs were used in PCR with appropriate primers (Table S1) by standard procedures. PCR products were analysed in 1% agarose. Chromosomal DNA was used with each set of primers to demonstrate amplification of a single specific region in each reaction.

Relative quantification of *cards* transcription was done by the comparative C_T_ (ΔΔC_T_) method in two-step real-time qRT-PCR using SYBR Green chemistry. Prior to RNA analysis, *cards*- and *pdhA*-specific primers (Table S1, MPN372P/M and MPN393P/M, respectively; the later served as a normalizer) were examined for specificity. Melting curves of amplicons confirmed generation of a single specific product in each reaction. Using primers with HeLa chromosomal DNA yielded no product. Validation of the assay was done using primer sets with serial dilutions of S1 chromosomal DNA. For analysis, 2 µg of total RNA was treated with DNase I (Invitrogen) and first-strand cDNA was synthesized using reverse *cards*- and *pdhA*-specific primers and SuperScript reverse transcriptase as suggested by manufacturer (Invitrogen). PCR reactions (50 µl) contained 25 µl of SYBR Green PCR Master Mix (Applied Biosystems), 2.0 µl of generated cDNAs (diluted 1:10) and corresponding primer set (1 µM each) and amplification was performed under default conditions using ABI PRISM 7900HT SDS (Applied Biosystems). All reactions were run in triplicate and plates included cDNA synthesis reactions without added reverse transcriptase (controls for DNA contamination). When analysing the mycoplasma response to co-incubation with HeLa cells, cDNA synthesis reactions performed on RNA isolated from uninfected HeLa cells were also included. Analysis was done using RQ Manager 1.2 (Applied Biosystems). *cards* transcript amounts are represented as a fold change with corresponding standard deviation.

Hybridization probes for slot blot analysis were also generated using the SuperScript first-strand synthesis system (Gibco-BRL). Twenty-five micrograms of RNA plus a mixture containing 0.2 µM of each gene-specific antisense oligonucleotide (Table S2) were heated to 70°C for 10 min and cooled to room temperature. Reverse transcription was performed with both [α-^32^P]dCTP and [α-^32^P]-TTP (NEN) replacing corresponding nucleotides in the reaction buffer. Reactions were heat inactivated at 70°C for 15 min, and *E. coli* RNase H (2 units per sample) was added prior to a 20 min incubation at 37°C. After unincorporated nucleotides were removed by gel filtration using G-25 Sephadex column chromatography (Roche), an equal volume of formamide was added. Denatured (boiling for 5 min) probes were added to hybridization buffer.

### Hybridization and data analysis

Blots were prehybridized for 4 h at 42°C in prehybridization solution [50% formamide, 120 mM Na_2_HPO_4_, 250 mM NaCl, 7% sodium dodecyl sulphate (SDS), 1 mM EDTA] and hybridized (prehybridization solution containing appropriate probes) at 42°C for 18 h. Membranes were washed at room temperature for 15 min in 2× SSC (1× SSC is 0.15 M NaCl plus 0.015 M sodium citrate)/0.1% SDS, 30 min in 0.5× SSC/0.1% SDS and 30 min in 0.1× SSC/0.1% SDS. After posthybridization washes, all membranes were sealed in plastic bags and placed in a PhosphorImager cassette.

Individual PhosphorImager screens were analysed using PhosphorImager 400E (Molecular Dynamics) with a pixel size of 176 µm, and resulting image files were assessed by determining pixel density for each band using ImageQuant (version 5.0) (Molecular Dynamics). Numeric files were exported into a Microsoft Excel spreadsheet for analysis. To analyse transcript amounts for different experimental conditions, we expressed values as percentage of *pdhA* transcript amount after background subtraction.

### Expression and purification of recombinant *M. pneumoniae* proteins and immunoblot analysis

Recombinant N-terminal His-tagged *M. pneumoniae* CARDS toxin, ClpB (Kannan *et al.*, 2008), DnaK, PDH-A, PDH-B, EF-Tu and carboxyl P1 truncation (unpublished data) were overexpressed in *E. coli* BL21(DE3) cells and purified using Ni-NTA (nickel-nitrilotriacetic acid) column chromatography (Kannan and Baseman, 2006). Verification of fusion proteins was performed by SDS-PAGE, Coomassie blue staining and immunoblotting with rabbit- or mouse-specific antisera. Concentrations of all proteins were determined by BCA protein assay (Pierce, Rockford, IL). For measurement of each *M. pneumoniae* protein, immunoblot analysis was employed using polyclonal rabbit or mouse antisera generated against *M. pneumoniae* CARDS toxin, ClpB, P1, P30, EF-Tu, PDH-B and *Mycoplasma genitalium* PDH-A and DnaK. To quantify protein expression by immunoblotting, a dilution series of each purified recombinant protein was loaded onto individual gels, probed with corresponding antibodies and signal strengths determined by densitometry. Quantification of band intensities was performed by scanning immunostained bands and analysing images with KODAK Image software. Using Microsoft Excel linear regression, we estimated protein concentrations in test samples, which were compared with standard curves. As necessary, samples were diluted so that the amount of loaded protein fell within the linear range of standards. Relative levels of protein expression were determined by comparing test unknowns to the 24 h sample as an internal standard. Transcript or protein amounts are represented in means with corresponding standard deviation.

### Separation of mycoplasma membrane, cytoplasmic and secreted proteins

In order to localize CARDS toxin in specific mycoplasma cell fractions, *M. pneumoniae* S1 cells were grown in SP-4 broth as described above. Cell cultures were harvested at 24, 48, 72 and 96 h by scraping and centrifugation, washed two times in 10 ml of 4°C Tris-NaCl [20 mM Tris-HCl (pH 7.4), 200 mM NaCl, 1 mM EDTA] and suspended in 1 ml of Tris-NaCl containing 20 µl of protease inhibitor cocktail (Sigma-Aldrich). Mycoplasma cells were disrupted by osmotic shock and sonication as detailed by us earlier (Dallo *et al.*, 2002). Then, samples were centrifuged at 100 000 *g* for 1 h to separate membrane and cytosolic cell fractions. Cytosolic fractions were precipitated with trichloroacetic acid at 20% saturation for 30 min on ice, suspended in 1 ml of Tris-NaCl and divided into 100 µl of aliquots or concentrated using Amicon centrifugal filter (Millipore; 3 kDa cutoff). Membrane fractions were further purified by sucrose gradient centrifugation to separate from unbroken cells. Then, enriched membrane fractions were suspended in 1 ml of Tris-NaCl, sonicated for 1 min, divided into 100 µl of aliquots, and membrane yield calculated relative to total cell lysate (Razin, 1983). To analyse secreted proteins, broth supernatants (500 ml) from mycoplasma cultures at each time point (24, 48, 72 and 96 h) were precipitated by ammonium sulphate in a three stepwise concentration (30%, 50% and 85%) (Englard and Seifter, 1990). Pellets from each ammonium sulphate fraction were resuspended in PBS containing protease inhibitors, dialysed against the same buffer, concentrated by Amicon centrifugal filter (Millipore; 3 kDa cutoff) and divided into 100 µl of aliquots. Membrane and supernatant fractions were stored at −80°C, and equal amounts of proteins were examined by immunoblotting for the presence of CARDS toxin (anti-rabbit antisera 1:2000), cytoplasmic EF-G (anti-rabbit sera 1:1000, gift from Dr Richard Herrmann) and membrane adhesin P1 (anti-mouse monoclonal antibody 1:2000). Based on immunoblot band intensities in each fraction and by membrane yield (constitutes ∼9–10% of the total protein), the percentage of membrane-associated CARDS toxin was determined.

### Immunogold electron microscopy of CARDS toxin location in *M. pneumoniae*

Immunogold labelling of *M. pneumoniae* cells was performed as reported previously (Dallo *et al.*, 2002; Balasubramanian *et al.*, 2008). Forty-eight hour cultures of SP-4-grown *M. pneumoniae* cells were washed with 100 mM Tris-HCl buffer (pH 7.5) containing 1% BSA and 1% heat-inactivated goat serum and incubated individually with anti-CARDS toxin, anti-P1 or anti-EF-G rabbit antisera at 1:100 dilution for 120 min at room temperature. After incubation for 60 min at room temperature with goat anti-rabbit IgG-gold (20 nm particles) complex diluted 1:40 in PBS, pH 7.4, with 1% BSA, *M. pneumoniae* cells were mounted on Formvar-coated nickel grids and fixed with 1% glutaraldehyde/4% formaldehyde for 20 min at room temperature. Individual grids were examined with a JEOL 1230 transmission electron microscope at 80 kV accelerating voltage after staining with 7% uranyl acetate followed by Reynolds lead citrate.

### Immunostaining and confocal microscopy to detect CARDS toxin in NHBE cells

For immunostaining of infected and control differentiated NHBE cells, airway cultures were gently washed with PBS and fixed with 4% paraformaldehyde (v/v) diluted in PBS containing 0.05% Tween (PBST). Fixed cells were permeabilized for 15 min with PBST containing 0.1% Triton X-100 (v/v), washed in PBST and incubated for 1 h at room temperature in blocking solution consisting of 10% normal goat serum diluted in PBST (v/v). Cells were then probed overnight at 4°C with mouse anti-CARDS toxin serum diluted 1:400 in blocking solution. The following day cells were washed with PBST and incubated for 1.5 h with Cy-5-conjugated anti-mouse IgG (Jackson ImmunoResearch Laboratories, West Grove, PA) diluted 1:75 in blocking solution. Cells were next washed with PBST followed by nuclei and actin counterstaining for 20 min using 1 mg ml^−1^ DAPI and 2 U ml^−1^ of Alexa Fluor 488-labelled phalloidin (Molecular Probes, Eugene, OR) respectively. After washing cells with PBST, membranes were excised from Transwell supports and mounted on slides containing Citifluor antifade (Ted Pella, Redding, CA). Optical sections of DAPI, Alexa Fluor 488 and Cy-5 fluorescence were imaged and reconstructed using a Leica TCS SP2 spectral confocal microscope (Leica Microsystems, Exton, PA).

### Animals and inoculation

Two-month-old mycoplasma- and murine virus-free female BALB/c mice (Charles River and Harlan) were housed in filter-top cages and allowed to acclimate to their new environment for 1 week. As detailed earlier (Hardy *et al.*, 2001), mice were intranasally inoculated once (day 0) with 2.5–7 × 10^7^ genomes of *M. pneumoniae* in 50 µl of SP-4 broth. Control mice were inoculated with sterile SP-4 broth. Animal guidelines were followed in accordance with the Institutional Animal Care and Research Advisory Committee at The University of Texas Southwestern Medical Center at Dallas.

### Mouse lung tissue collection and analysis

Lung samples were obtained at 1 and 2 days post infection. At each time point, infected mice and SP-4-inoculated control mice (five each) were utilized as the source of specimens. Frozen lungs were placed in PBS containing protease inhibitors (Roche), precooled to 4°C and disrupted for 3 × 90 s at 20 Hz in the presence of 5 mm stainless steel beads using TissueLyserII (Qiagen). Resultant lysates (50–100 µg) were used to evaluate the number of *M. pneumoniae* genomes and CARDS toxin protein levels. Lung samples from both control and infected mice at different time intervals were subjected to DNA isolation, and mycoplasma genomes were determined by qPCR using P1 adhesin (MPN141) sequences as described previously (Ursi *et al.*, 2003). Standard curves were established using *M. pneumoniae* S1 chromosomal DNA serially diluted from 10^7^ to 1 copy per reaction. To confirm the absence of interference and cross-binding, standard curves were evaluated with DNA isolated from tissues of non-infected mice. All reactions were performed in triplicate using TaqMan Universal PCR Master Mix, 0.5 µM concentration of *M. pneumoniae*-specific primers and 0.2 µM concentration of appropriate probe. Amplification conditions consisted of 50°C for 2 min, 95°C for 10 min and 40 cycles of 95°C for 15 s and 60°C for 1 min. Real-time PCR was done using ABI PRISM HP7900 SDS (Applied Biosystems).

To quantify the amount of CARDS toxin in individual samples, toxin ‘antigen capture’ assays were performed. Flat-bottom, 96-well microtitre enzyme-linked immunosorbent assay (ELISA) plates (HBX 4, Dynatech, Alexandria, VA) were coated with 50 µl of rabbit anti-CARDS toxin IgG (10 µg ml^−1^). Plates were blocked with 3% BSA for 2 h at room temperature and washed with PBS containing 0.05% v/v polyoxyethylene sorbitan monolaurate (Tween-20, Sigma-Aldrich) (PBST). Ten microlitres of extracted lung sample or SP-4-grown *M. pneumoniae* cells was mixed with 1% BSA/PBST to a final volume of 50 µl and added to individual wells in triplicate. In order to quantify the amount of CARDS toxin in a given sample, known amounts of highly purified CARDS toxin (ranging from 10 pg to 10 ng well^−1^) were added to separate anti-CARDS toxin-coated wells and run in parallel in each ELISA plate. After 2 h incubation at room temperature, wells were washed with PBST, 50 µl of 1/2000 diluted affinity-purified HRP-conjugated anti-CARDS toxin IgG (0.5 µg ml^−1^) in 1% BSA/PBST was added, and plates were incubated at room temperature for 2 h. Plates were washed as before, and 50 µl of enhanced K-blue TMB substrate solution (Neogen, Lexington, KY) was added to wells. Incubation was continued for 10 min at room temperature until reactions developed colour. Assays were terminated by adding 0.05 ml of 1N HCl. Optical densities (OD) at 450 nm were determined in an automatic ELISA plate reader (Dynatech, Alexandria, VA) and buffer without test material served as background OD_450_ = 0. Data are expressed as mean ± SD from at least three to five animals from each time point of two experiments except where otherwise indicated.
