# Supplementary material for: Mycoplasma pneumoniae Community Acquired Respiratory Distress Syndrome toxin expression reveals growth phase and infection-dependent regulation
Source: Mol Microbiol. 2010 Mar 3;76(5):1127–41. doi: 10.1111/j.1365-2958.2010.07092.x (PMC2883071; doi:10.1111/j.1365-2958.2010.07092.x)
Supplement: Supplementary file 1 [file mmi0076-1127-SD1.pdf]

Fig. S1

```
   1  TACGTAACGCGTAAAGAGTTCAACGAGTACAAAGACTCAAACGACCAACGACTTACAAAG
  61  ATTGAAAATAAGGTCGATAAGCTTGAAATTAAAGTTGATAAGCTAGAGAAGAAAGTCGAT
 121  AAGCTGGAAGTTAAGGTTGATAAACTAGTCGAAACTGTTAATGCTCAAGGAGAAGATCTT
 181  AACAACTTCAAAGTTGAAGTGAGAGGAACACTTCAAAGCCAAGGCGAAACCCTGCAATTA
 241  ATTTTGCAAACGCTACAAGGCATGAGTAAGCGGCTTGATTCTGTTGAAGGACGTTTAGAC
 301  TCTATGGATGGTCGTCTCGATTCAATGGAAACTCGTTTGGACAAAATCGATCCGCTAAAA
 361  TAGTCTTAGGCTAATAACAAAAGTGCCAGCAATTAATCTATAAAGGTTAGTCGCTGGTAT
 421  TTTCATTTATTTGAAATAAATAAAAAAGCCATCCTATCAAGGAGAAAGACAGACAGCTAA
 481  TGAATTGAAATCAATTACTTAATTAAATATTCCAAGTAAATGAAGAGATTAATATCTGAT
 541  GAATAGCCTTCTGTATTATTAGCAAAGGTACTTAAAAATTTGTTCACACTTTTTTCACTA
 601  ACATCATCTTTGCCTTTTAATGAAAAGCTAAGTGAAGCGCTAAACTCAATTCACTCTAAA
 661  TTTCTTGTAATTTCTTTTCCATTAACTTTAATTTTTTCTTCTTTAGTAAGAAATTTTTCT
 721  GTAGTAAATTGTTTTCCTGGATCAGAATTAGATTTAATTTTAGGGAAAAGATCAAAATAA
 781  TGCTTGTCACCTTTCTTAGCAGTGAAATCACCTGAAGAATAATCTCCAAACCAATTAAAG
 841  GTATAGCTCAACTTAGCTTCAATTGACAAATCCTTAAAACTTTGGTATGTCATCTGGAAA
 901  TATTCAATCTTTCGCTCCATCGTAAAAGGATCCTTGCTTGCGGGATAAATTACATCGTCA
 961  AAATCAAAATCTGGAATACCTAATTCGCCCTTTTCCGCACTTTGTTTTCAGGCGTCTAAC
1021  AACAATTTTGTTGTATCTTTCGGATTAACTTTGTAAGACTTCTTTAGAGAAGTTATCATG
1081  TTCTTTTTGCCTTCTGTAGTTTTAATTGCTTTATACACATCAATGTCTTTACCGGTGGCT
1141  TCTTTAATAAGATTTCTAAGATCTGCTTGGATACCAGTACAGGCGCTTAAAACTGTTCCA
1201  CATAACAAAAATAAAGAACCAAAGGAAACCTTTGACAAAAACTTCTTTTTGCGCATCAAC
1261  AGTAAAAATAACTCTTTAATTTTGCTTTTTTTGTAAAAAGCCTTTTTGGGATAAGAACTT
1321  GAATTGGAATTTCGGGTTGAACATTAATTAAAACACTGAAATTTCCAATCATTTATTAAC
1381  AACTATCTACTGATAAAGGTTTTGATCTGAGTAGTTCTAATAATTGCTAATTGTTGGCAT
1441  TTTTATTCATTTAATGCTTTCTAAAAACTTTTTGCAATTTTGTTGGCACTTCTCAATAAG
1501  TCGGTCAATTTATTTAGTGGAAGAGCAAATGCAAATCTTTAATTTTTTGAATTGATCAGC
1561  AAAGCGTCTGATCCCTGTTGGCAACCGAGTTGCAAGTGTTAGTTATTTTTTAAAAAATCA
1621  AAAGAAAAAAGAAAGGAACATTTAAACATGAAAAAAGTTAAAGCATTAAGGAATCTTTTT
1681  CTAGAAAAAGCCAATGAAAAACTTTAATACCCCAAAATTGCTTCAATGGTAGCTTACTTT
1741  GATCCAAAGGAAGACGGTACTTGTGAATTCATTATGAAAATTAAAACTGTAAATGAAGTT
1801  GCTGACACTGAGATAACAAATAAGGAAAAGCCTTTAGGCACTTGAAAGAGTTAGTTAACT
1861  TTTTGGTCTAAAAAGACATAAATTTACCAACGGTAAGTGTTAAAACTTGCTGTTGGTATT
1921  TTTATTTCCCCCACGCTCACCAGGTTTAGTACCGGGTTCAACCTCGCGGGGTCGGTGCTC
1981  GACCAGGTGTTGGATTATGTGCCCTGGATTGGGAATGGGCACAGGTATGGGAATAACCAC
2041  CGGGGCGTGGATGATATAACCGCGCCTAAAACCGGCGCGGGGTCGTCCAGCGGAACTAGT
2101  ACGAACACAAGTGGTTCGCGTTCCTTTCTCCCGACGTTCTCGAATGTCGGCGTTGGCCTC
2161  AAAGCGAATGTCCAGGGCACCCTGGGTGGCAGTCAGACGACGACTACGGGGAAAGATATT
2221  CCCAAATGACCCACCCTCGACCCAGCCAACCTCCAGCTCTGAACGGGGGCGGGGTGAAGG
2281  AATGATAAGGCTTCAAACAAACAAAGTGACGAAAACCACACCACCTTTAAAAGCGCTACG
2341  GGCAGTGGCCAGCAGGGTGGTTCTACAACGGGTGGTTCAGCGGGCAATCCCGGCTCGTTA
2401  AAGCAGGATAAGATTAGTAAATCAGGTCAGAACTTAACCACGCAGGACGGCGCGCCCCAG
2461  TCTAATTCGACGACGGAATCCGCGTCGAATTATGATCACCTCCCCCCAACCTCACCCCCA
2521  CATCCGATTTACCGAACGCGCTGTCATTCACCAACAAGAACAACGCGCAGCGCGCCCAGC
2581  TCTTCCTCCGCGGCTTGTTGGGCAGCATCCCGGTGTTGGTGAATCGAAGTGGGTCAGATG
2641  ATTCCAACAAATTCCAAGCCACCGACCAAAAATGGTCTTACACCGACTTAAAGTCGGACC
2701  AAACCAAGCTCAACCTCCCCGCTTATGGTGAAGTGAATGGGTTGTTGAATCCGGCGTTGG
2761  TGGAAACCTATTTTGGGACGACGCGAGCGGGTGGCTCGGGGTCCAACACGACCAGTTCAC
2821  CCGGTATCGGTTTTAAAATTCCCGAACAAAATAATGATTCGAAGGCTGTGCTGATCACCC
2881  CCGGGTTGGCTTGAACGCCGCAAGACGTTGGTAACCTCGTTGTCAGTGGTACCAGCTTGA
2941  GCTTCCAGTTGGGCGGGTGGCTGGTCACCTTCACGGACTTTGTCAAACCCCGCGCGGGTT
3001  ACCTCGGGCTCCAGTTAACGGGCTTGGATGCAAGTGATGCGACGCAGCGCGCCCTCATTT
3061  GGGCCAAGCAGCCCTGAGCGGCCTTTCGTGGCAGTTGGGTCAACCGCTTGGGCCGGGTCG
3121  AGAGTGTGTGGGATTTAAAGGGGGTGTGACAAGATCAAGCTCAGGCGGCCGCGCAAGCAG
3181  CTACCACCACCGCCGCAACAGGGGACGCCTTACCGGAGCACCCCAATGCCCTCGCGTACC
```

3241 AAATTAGCTCCACCGACAAGGATTCGTACAAGGCTTCCACTCAAAGCTCCGGTCAAACCA
3301 ATTCCCAAAACACCTCCCCCTACCTCCATTTGATTAAACCTAAGAAAGTCGAAAACACGA
3361 CCCAACTCGACCAGGGCTTAAAAACCTGTTGGACCCCAACCAGGTTCGCACCAAGCTGCG
3421 CCAAAGCTTTGGTACAGACCATTCCACCCAAGCCAAACCCCAATCCCTCAAAAAAACCAC
3481 ACCGGTGTTTGGGACGAATAGTGGTAACATTGGCAGTGTGCTTAGTGGTGGGGGTGCTGG
3541 AGGAGCAGACAGCACCAATTCGGTGGACCTCTCCCCCGTTGAACGGGTGAGTGGGTGGCT
3601 TGTGGGGCAATTACCCAGTGGGGGTGGGGGGAATAGTAGTGAGGATATTAAAAGTGTGCA
3661 AGACACTCCTATTTATGATCTGTATTCATTAGTATATTCTTTTTAAATTGTTCCCTCACT
3721 TTATTCATATGAACCACCGCCTCCCTCGCGACGGGACTCACCGTAGTGGGACACTTCACA
3781 AGTACCACCACGACGCTCAAGCGCCAGCAATTTAGCTACACCCGCCCTGACGAGGTCGCG
3841 CTGCGCCACACCAATGCCATCAACCCGCGCTTAACCCCGTGAACGTATCGTAACACGAGC
3901 TTTTCGTCCCTCCCCCTCACGGGTGAAAACCCCGGGGCGTGGGCCTTAGTGCGCGACAAC
3961 ACCGCCAAGGGCATCACTGCCGGCAGTGGCAGTCAACAAACCACGTATGATCCCACCCGA
4021 ACCGAAGCGGCTTTGACCACCGCCACCACCTTTGCGTTACGCCGATACGACCTCGCCGGG
4081 CGTTGTACGACCTCGACTTTTCGAAGTTAAACCCGCAAACGCCCACGCGCGACCAAACCG
4141 GGCAGATCACCTTTAACCCCTTTGGCGGCTTTGGTTTGAGTGGGGCTGCACCCCAACAGT
4201 GAAACGAGGTCAAAAACAAGGTCCCCGTCGAGGTGGCGCAAGACCCCTCCAATCCCTACC
4261 GGTTTGCCGTTTTACTCGTGCCGCGCAGCGTGGTGTACTATGAGCAGTTGCAAAGGGGGT
4321 TGGGCTTACCACAGCAGCGAACCGAGAATGGTCAAAATACTTCCACCACCGGGGCAATGT
4381 TTGGCTTGAAGGTGAAGAACGCCGAGGCGGACACCGCGAAGAGCAATGAAAAACTCCAGG
4441 GCGCTGAGGCCACTGGTTCTTCAACCACATCTGGATCTGGCCAATCCACCCAACGTGGGG
4501 TTCGTCAGGGGACACCAAAGTCAAGGCGTTGCAGGTGGCGGTGAAAAAGAAATCGGACTC
4561 GGAGGACAATGGTCAGATCGAACTTGAAACCAACAACCTCGCCAACGCCCCGATTAAACG
4621 GGGCTCCAATAACAACCAGCAAGTCCAACTCAAGGCGGACGATTTTGGTACTGCCCCTTC
4681 CAGTTCGGGATCAGGCACCCAAGATGGCACCCCCACCCCCTGAACGCCGTGGTTAACGAC
4741 TGAGCAAATTCACAACGACCCCGCCAAATTCGCCGCCTCGATCCTCATTCTGTACGATGC
4801 GCCTTATGCGCGCAACCGTACCGTCATTGACCGCGTTGATCACTTGGATCCCAAGGTGAT
4861 GGTGGATAATTACTGTTAACAATTTTTTTAGAAAAAGGCAGTTTTTGAAGTTTTTAATTT
4921 GTAAAATTTCATTTTTTAAAAATGCCAAATCCTGTTAGATTTGTTTACCGTGTTGATTTG
4981 AGAAGCCCTGAAGAAATTTTTGAACATGGCTTTTCAACTTTAGGTGATGTGAGAAATTTC
5041 TTTGAACACATTCTCTCCACTAATTTTGGTAGAAGCTATTTTATTTCCACTTCAGAAACA
5101 CCCACAGCAGCTATTCGCTTCTTTGGTAGCTGGTTACGGGAATATGTACCAGAGCACCCC
5161 AGAAGGGCTTACTTATATGAAATTCGTGCCGACCAACACTTTTACAATGCCCGCGCCACT
5221 GGGGAGAACTTGTTAGATTTAATGCGTCAAAGACAAGTAGTATTTGACTCTGGTGATCGA
5281 GAAATGGCACAAATGGGAATTAGAGCTTTACGCACTTCCTTTGCGTATCAACGTGAATGG
5341 TTTACCGATGGTCCAATTGCAGCAGCTAATGTCCGTAGTGCTTGACTAGTAGATGCTGTT
5401 CCCGTTGAACCTGGTCATGCTCACCACCCGGCTGGTCGTGTTGTAGAGACTACTAGAATT
5461 AATGAACCGGAAATGCACAACCCTCATTATCAAGAGCTGCAAACCCAAGCCAATGATCAA
5521 CCATGATTGCCAACACCAGGAATAGCTACTCCTGTACATTTATCAATTCCCCAAGCAGCT
5581 TCCGTTGCTGATGTTTCGGAAGGTACTTCCGCTTCGCTATCGTTTGCGTGCCCTGATTGA
5641 AGTCCACCTTCTAGTAATGGTGAAAATCCGCTAGACAAATGCATTGCGGAAAAGATTGAT
5701 AACTATAACCTACAATCCTTACCACAGTACGCTAGCAGTGTAAAGGAACTGGAAGATACA
5761 CCAGTATACCTAAGGGGAATTAAAACGCAAAAAACCTTTATGTTACAAGCAGATCCGCAA
5821 AATAACAATGTCTTTTTGGTCGAAGTAAACCCCAAACAAAAGTCCAGCTTTCCCCAAACC
5881 ATCTTCTTTTGGGATGTTTATCAACGAATTTGTCTCAAGGATTTAACTGGTGCACAAATC
5941 AGTCTTTCGCTTACTGCCTTTACTACTCAGTATGCTGGTCAGCTCAAAGTGCACCTTAGT
6001 GTTAGCGCGGTTAATGCCGTGAACCAAAAGTGAAAAATGACACCGCAAGACAGTGCAATA
6061 ACTCAGTTTCGGGTCTCCTCTGAACTGTTAGGTCAAACTGAAAATGGCTTGTTCTGAAAT
6121 ACCAAGAGTGGTGGTTCACAACACGATTTGTATGTATGTCCTTTGAAAAATCCACCTAGT
6181 GATTTGGAAGAATTACAAATAATTGTTGATGAATGTACTACCCATGCGCAGTTTGTTACT
6241 ATGCGTGCAGCTAGCACCTTCTTTGTTGATGTTCAGCTAGGCTGGTATTGAAGGGGTTAT
6301 TACTATACCCCACAATTAAGTGGTTGATCTTATCAGATGAAAACACCAGATGGACAGATA
6361 TTCTATGATCTAAAAACTTCGAAAATCTTCCTTGTCCAGGACAACCAAAACGTGTTCTTT
6421 CTCCATAATAAACTCAACAAACAAACTGGTTACAGCTGGGATTGAGTAGAATGGCTAAAA
6481 CATGACATGAATGAGGACAAAGACGAAAACTTTAAATGGTACTTTTCGCGTGATGACCTT
6541 ACCATTCCTTCCGTTGAAGGGCTTAACTTCCGCCACATTCGCTGTTACGCTGACAACCAG
6601 CAGTTAAAGGTGATCATAAGCGGTTCACGTTGGGGCGGTTGGTACTCCACTTACGATAAA

6661 GTTGAAAGTAATGTCGAAGATAAGATTTTGGTCAAAGATGGTTTTGATCGCTTTTAGCGA
6721 TTAAGCTTTAACGTCACTGTTTTGCTCTAATGTTAGAAGCAAAGATCTTGTCGACAAACT
6781 CGTTGCGCTTGCGGTAACGACGTACTACTAAATAAGTTACAGCACCAGCAGCTACTACAC
6841 TAGCTATAGCAGCCACAGTTAAACCAGCTACCAACGGATCTACGTGGGTTAAGAAGTTGA
6901 AACTGCCACCATTTGCATTCAATTCAACGGTAAAGCCATCTAGTGATCTTTCACCTTGAA
6961 CTAGTTGTTGGCTAGACAATGAGCGTGGTTTGAAGAGGTCAAATGAACCATTAGCACCAA
7021 CTTGGTTTTCGTACTTAACTTGTAAGAACTCCTTCAATTGTCTTTGGAAGGTACTTTGAA
7081 TTGGTGCATAAGCATCATTAATAAATGAGCTGTCGGTAAACAATGCCCCTACAATCATGT
7141 TGTACTCATACGGATTGAGTCCAGAGAAATCAAACACAAGTGTTTTCTGCTCCTTATTAT
7201 TTCCCACACTTCTATTTTCTATTTTGTAAGGAATATCAACCGTAGTGCTTTGGTTTGGGT
7261 TGAATGCATTCTTAGTTAAGGCAATTCTTAGATTACCACCTTCAGCATTATTGTCAGTTT
7321 GGGCACCGCCATCACTTACCATAACTGTGTAAGATTGTGCTTCCTGTGCCAATTTGTTGA
7381 CATCATTTGGGTCATTCAAACCTAAAGAGCCATTTTGTTCAAGCGAGAAGTCGGCTTTTA
7441 AGTCTAGCTTGATGCCATCCTTTTCAACATCAAACTTAATTTCGTCCTTTAAGAAAACCT
7501 TCTTGTTAGTTAGTCTCTCTACATAACCAACCACCGCACCACTAGCAGCTTGACTTAAAA
7561 CACCACGTTTAGAACTCTCGTTGGACTTCAAGAAGTGCGCTACGATCTCTCTGTTTTTGG
7621 TTGAAAGAGAAGTGGTTGTTTGGAAGAAGTTTTGCGGTAATTTTCCCCTTCAATCACTAG
7681 TGTTGAAATACAGTTTAGATTTTTGTCTTGCTTGAATTACAGCGTCCCTTGAGGCATTAT
7741 CGACGTATATACCGTTGTTGTCGAACCTAAGTGCATCGAGTGAGTAGAACTTATCCTTTG
7801 TTTCAAAACGGATAATGTAAGTACTTTGCGCGTTCTTGTCTAAACCTTCTAGGTAATAGA
7861 AATAGTTTCTAGGGTTAGCAATACGACGTTGGTTACCAAGGTTGTTAGCGTTAGTTACAT
7921 CTACTACCACATAGAAGTGACCATTGCTTTCATTTCCGTTCAAGTTAATATTGTTCTTAG
7981 TTTGAAAGTTTTGCAGTGCAGTTTTGAGTTGAGTATCTTTAGTATCAGTGCTGCCTAAAA
8041 CATCTTGAACTTTGCGTACGTTATCAAAGTCGAGGTTAGTGCTAATACCGTTGCTGGTAA
8101 GGTAATCGTTAATGCTCTTGTTGAAACTCTTGTCATCAATTTCACGTAACTGATCATTTT
8161 GTGCTTTTCAAACAATACTTCTTGTCTGTAAGTTTGCCCGTTCTCAACTAGCACTTTGGA
8221 AATCTCTTCTTCAGTTAATATCTGAAGACTAGCTTGTTGTCTTCCTTCTTAAGTAGTGTT
8281 TTAGCTACAGATACAACCTTATCTTCTTTCACTTCCAAGCGATCAAGGAAGTCATTATCA
8341 CGTCAGCCACCATCTTGAAAACCAATTGCATTAACAATAATTTCAGCTTTGTTTTCGTTT
8401 GCTAAAGCATTGAGCTTGGTCTTCATTGTTTTCAATTGCTAAGAACTGGAAGTTTTGTCC
8461 ATCCCACACTTTAACTTCCTTAGTTACTTTTTGTCCTAGGGTAACGTTAGCGTAAATGGC
8521 CATGGAAAAGTAAGAAGCTACTAACTTTAAGAATTGATCTCAGTCCTTCGGTTCTTGGTT
8581 TAAATTAAGACCTAATTCCTTATTTGCTTCTTGATACCACTTAACTCAACCGGTTGAGTT
8641 AATTGGAGTACTTACTTTATTGGTATCTTTATCTAGGCTACTAATGTATTCTTTTACCTT
8701 TAGGTAATCAACTTGCTTGTTTTTAATTTGCGTTAAAACGCTGTTAATGCCAGCTTTTTC
8761 ACTTTCGTTACTTTTGCTCTCTAAACGTTGTTCTAAATCCTTTACTTGTTCAGAAAGGTT
8821 GTAAACGTTAGTGATTGCATTAACAAAAACTGGATTTAAAACAAAATCTAAGGTAGGTGC
8881 TTGAGTTAAAACACCGTTTCTAGTTGAATTTAGATTAGCGTTTGTTTTAGGGATCAACAG
8941 CTTATCTAAGTTAAAGTTTTTGTAACGGACAAAACTAAAGCTTTGGTTTTGTAGTTTGTA
9001 ATTTCTGTTTTCAAACTTAAAGTTAGTTCCAGTGGTGTTAGTGTCAATCACCTCTTTCAC
9061 TGCACTACCTAACCCAACATTTAGAGTTTCATCCTTGCGTACGTATGCTTTAAGTTGACT
9121 TTCAGCTGCACTTTGATAGTTGGCCTGTGTACTCTTAGCGTTAGCAATAAAGTCAGCTGT
9181 TTTTATCGATTGCTGCTGACCATTGCTGTCGGTGTAAAAAAGCTTGGTTCAAGGATCTAA
9241 CTTAACAAAGAAAGTAATGCCGTGATTAGTAAAGCTCTTTAAAGGACGGTCGGAATAATG
9301 AATTGTTGCAGGGTAATCTAATGGATCACTGTTAGCACCTAAGAAAGTGGTTTCAAAGTT
9361 TACGTTAGGTTTTTCAATGAAGTTACGATCTTTGTTTGCAAGTCACTAGTTTCTAGTAGT
9421 TGTAAAACCAGATTGACGGAACCATAAGAATACTCCTTTCTCTGAGTGGAGGTTGTTGAC
9481 GTTTTGTAACAGCTTTTCTTTAATGCTACCCCAAACGGTTTTGGAAGTTTCGTTTGTTGC
9541 GGGGTCAGATTTTTTTAAATCTGCTTGACGGTGAAGTTCACCCCTTTCAATGAGCACTAT
9601 CTTTTGGTGGGCAAAGTCAAGGTCAAACCAAGCAGTTCATCTCCATCGATCATCATTGGT
9661 TCCAGCTTTTTTTGCACCCCGGTAGTGGGTTTTGTAATACTTGAACTTATCATACCCTGC
9721 TCGGTATTTTGTTTCATGTCGAAACGGCGATTTCCAACCGTTATTATTAACAGTGCCCAC
9781 TAAACCAAAACTCATATCTGACTTAGTTTAGATGTTTACTTCTTTTATTCCTGGAAACTT
9841 GTTACTTAATTTAGAAAAGGAAGATCCACCAACAAGCCAACCAAAACCAGCGAGTCCAGC
9901 ACACAACAGCAACGAATAATTTCGTAAACGCTTTATCCTCATACGTGTAAATTTATAACA
9961 AAAAAACGCCCCTTGGGGAACGTTTTTAAACAAGTTTAATTATTAGTCATTTTTATTGAA
10021 TATTAAATCTTCATTTATTTAATGCTTAAAGATTAATTAACTTTTGCACTTGCTTAAGAA

10081 GTCCATTTTGACAAAGAAAAATCCCAAAAGGATGGCAATAAAGCTCACGCTTTTGGGATC
10141 TTCCTTGTGGTTTAAACCATTTTTTTAGCTAAGTCTACTTAACTTTTCTTGCTTTGAGCA
10201 TTTGTTCTCAGAAGTGCTTACGACTCTTACGTCAGCGGTGTAAGACAACACCACTAGCTA
10261 TTATTGTTGAAAGTAATGCAGCACTACCAGTGAAAGTAATTGCTATTAATGGTCAGTTAA
10321 TCTTATTGATGTCTTCTTTATTGAAAATGTTTTTGTGTAGATCAAAGGTGAAGTTATCAA
10381 TTTGTTTTTGGTTATTTGCCAATTGTACCTTGGTCAATAAAGGTAGTTGACCAGTCTTAA
10441 TTGGTGCTTGGTATTTGTGTTCTAAAACGGTTTTGAGCTGCTTTCTAAATTCTGTTTGCA
10501 ATGGAGCGTAGGCATTCTTAATAAAGGAGCTGTCGGTAAACAACAGACTAACAACTGTGT
10561 TGTATTCGAAGTTGTTTAGGTTAGAGAAGTCAAACACCAAGTTTTTCTGCTCTCTAACCG
10621 TACCATCCTTGTTAGTTTTGGATTTCTTGGTAACAATCCAGTAAGGCAGTTTAATAACGG
10681 TACTTTGTTCAGGGTTGGTGGTTAAAGTAATCTTGAAGATGTTTTTGTCTGATTCTGGAT
10741 CATCACCTTTTTCTTCTGAAACCAAGACATTGTAACCCTTAGCTTCTTCTACAATTTGGT
10801 TAATTACACTTGGGTCTTTAACCTCTAAATTACCCTTCTTGTCTAAGGTAAAGTTCGATG
10861 TTAAACGTAGCTTTAAATTATTTTTGTCTTCCTTATTAAAGCTCACCCTTTCCTTTAAAA
10921 AGAGCTTTTTACCAGTCATTCGCTCAACATAGCCTAAGATCGAACCACTGACATTTTCAC
10981 TCAAAAAACCCTCATCATTCTCAGCGTTAGGTGTAGATAGTTTAGCTGAATTGTCAGCAC
11041 TTGCTGTAGGCAAAGTAAGTTCAGCGTTGGTGAGGTTAGCCTTCAAAGCAGCGTTCCAGT
11101 TATGAGTATCGAGATAAAGGTTTTGGTTTTGCTTGGCTTGGATAATTGCATCCTTGGACA
11161 CATTTTTCACATAAAGGCCACGACTATCAACCGCTAGTGATTCGAGTGAATACAGCTTTT
11221 GTTTGTTTTCAAAGCGCACTAAGTACGAACTTTGTGCTCCTTTGTCCAACCCTTCAATAT
11281 AGTAAAAGTAACTCTTAGCGTTATTTGCCCGGCGTTGGTTACCCAAGTCATTGGCATTAG
11341 TTACATCCACTACTACATAAAACTGACCAGTGTATGGGTTAGATCGTAGGTTTAAACCAT
11401 ACTTGTTTGCCGTACTTTGAACGCTAGCGAGCTTCGGATCATCACTTTTGCGGGCGTTTA
11461 ACATCGCATCAACTTTATGAACAGCATTTACTTTAATTTCAGTTTCAGAGATGCCTTGTA
11521 CCTTTAAATAGTTGTTAATGTCCTTTTGGAAGCGTTCGCGCTCATCGTTCAAATCAGTGC
11581 TTTGTACTACAATACTCTTGGTTTGTGAGTTTTGTTTGTCCCAGCTAGCTTGTTTAAAGT
11641 TTTGTCGGTAATGGTACTTATAAGAAATTTCGTTGGCTGCTTTTTCTATTTTGCTAGCAT
11701 CACCGTTAGCAATTTTAGTTTTACTACTCTTAGGGGTTAGAGCTACCTTATTGATAAAAC
11761 TAGCATCACGTAAACCGCCTTCATCTTGAAAACCAATGGCGCTTACAGCAATCTCAGCGC
11821 GGTTTTCATTTGCTAGTTGTTCTGATTGTTTTTCAGTGTTTTCAATCGGGATGAACTCAA
11881 ACTTAGCACCATCCCAAACCTTCATCCGTCTTTCCTTTGCGCCGTTTAATTTGACGTTAG
11941 CAAAAATGTCCTTAGAGAAGTAAGAAGCAATTAAGCTGGAAAGTTGTGTTCAGGTATTAA
12001 CATCATCGTTCAAGTTTAAACCAAACTCTTTTTTAGCATCAGCATACCATTGTGCAGTAT
12061 TTTCACCCTTGGATTCAATACTACCTGGATTTTGGCTTCAATTTTTCAGCTTGCCAATAA
12121 AATCAGCTACTTGAATGTAGTCAACTTCCTTATTTTTGATTTGTTCTATAACCTTATTGA
12181 TTGCTGCTTTGTCACTTTCATTGTAAGGTTGATCAGACTTTAAATGCTCCTTTAAATCAA
12241 GTGTTTGCGCAAACAGATTGTGAATGTTGACAATGGCGTTTGTCAAAACGGGGTTAAAGT
12301 CAAAGGACAGTGTTGGTTGTTTGACAAAAACACCATTCTTAATTGCCGTAGCATCCTCGT
12361 TGGTTTTTGGCACTAGTAACTTATCGAGCTTAAAGTTATTGTAAGTGTCAATGCTAAAGG
12421 TCGAATCCTTAAGTTTGTACTTTAGCTGTTGGTTGGAGGCTATATCACTCTTAGTGGTGT
12481 AAGTTTGACGTACAGCACTACCTAAACCTAAGTCAATACTGCGAGTTTTTCTAACAAGCG
12541 TTTTAATCTGGTTTTTATTAACCCATTGGTAATTAGTCTTGGTACTCTTTGCTTTTCTAA
12601 TGTAATCACTTACAGTAATTAATCTTTCCTGACCGTTTTCCTTGTAAAACAGCTTGCTGT
12661 AAGGTTCTAGCTTTAAAAAGAAGCGCGTGTCAGTGGTATGACTACCTCAATCAGGACTGT
12721 GCCAATTGGAGTTACCACCTTCAGAAGTGATGTTGGTGGTAAAGGCATTGGGGTTATTGT
12781 TGGTATCCCAAAAATCGGTAAAAAAGCCACTGTTAGCCCAGCCCTTAGTACCCTTGGTGT
12841 TAAAACCAGTTTGGTTGAACCAGATGAAGATTCCCTTATCTGGATCTAACCGCTCAGCAC
12901 TGTAGAGAATCTTCGAGCGAATATCACCCCATAAAGGTTTGTTTTTATCCCATTTTCTGG
12961 GAGTTGGTTCTGGAACACGCTTGTCAATATGATATTCATCATTTTCCACCAAACCTATGC
13021 GTTGGTTGGCATAACCCAAATCAAACCAAGCCACCCACTTTCAGTAATAAGGGTTACTTG
13081 CTGTCCTTTCGCGGTTAGAGTACTTGTAATATGTTTGGTTCCAGCTAAAGTGAACGTTTT
13141 TGTCGTATTTGTTCTCGTGCCGGAACGCTCAAGTGCGACCGTTATCAGTGTGTCAACCCA
13201 CTAAACCAAAGCTAGCGCCAGAGTTGGTATACACCTTTACATCTGGTAGTTCACCGTTCT
13261 GAATGGAAAGCTGAGCAGTACGCTCAATTTGCTTAAGTGCTTCTTGAGCGTAAAGGCTTT
13321 CACTAATGTAGTTATTAACGTTGTTTAAGTAGACCTCTGTCTTTTTACCGTTATTTCACT
13381 TTAAATCAAAGCGCTGGGTAATAGCTGGACTTTTACTCTTTAAGATTGAAAGCACATCGC
13441 CTTTACCCTTAAGGTAGCGCATGTGAACGCCACCATCGCTTGATAGTGTTTCATTGTTAC

```
13501  TTTTAACAACGCTAGTGAATGCTGAACTTGCAACGAATCAACCAAAACTTCCTAATGCAA
13561  AACCAAAAAGTAAGAATCGGTACTGTAAACGTTTCATGCTTTATTCTAGCACTTTAAAAA
13621  TATTTTTGACAATCCTTCTGCGCGTGTATTAAAGCAAATGCTGAACGAAATTGACAAAAG
13681  ACGCACGGTTTGAACTCAAAAAGCTTTTTATGGCGTGGTATTTCACCAAGCATTTCATTC
13741  ATTTTACCCTCTAAATGAAGAGTTACTTTGCTCTGCTTATCTCAATATAGTTAAAGTAAT
13801  GTGTATAAACGTAGCTAAAATAAAAAAACCAACTGAGAGCAGTTGGTTGGAATCAATAAT
13861  TTGGCGCGCCCAATAGGACTTGAACCCATAACCTTCTGGTCCGAAGCCAGACACTCTATC
13921  CAATTGAGCTATGGGCGCTTCTTATAGTTAATTCTATTAAATCTCTGAGAACTGCGCAAA
13981  TCTTTATTAAAATTAGCTGACTTCAACACAGTCGCTCTAATACATGTCAAAGGATAAAAA
14041  AAATAAGGTTGAACAGCTTGAACCAGTAGACCTTTTTGAGCGGACTAAGCTAGAGGACAC
14101  CCAAGTTTTAAATGATGTTGAACTAGATGACATCAAGAAACTAGAAGAGTTGAAAAAAGA
14161  ATTGGAAAACACCTTTGATCCACGTACCAGAATTGAAATTAAACGTGAAATCAAAGAGTT
14221  GGAACGTAAACTAAGAAGGAACCGTTAGTGAATGTTTGTTAACCTCCACACTAACTCTTA
14281  CTATAACTTCCTCAACTCCACACTTTCACCGCAAAAGCTAGTTGACCTAGCTGTTCAAGA
14341  CCAACAGGTAGCTGTTTGTTTAACCGATCCCAACTTATTTGGTGCTACCGAGTTTTTTTT
14401  GGCCTGTCAAAAAGCGCACATTAAACCCTTAATTGGGTTGAGTGTTACTGTTCGACATTA
14461  CGAACAAAACGTTAATTTACTAGTAATTGCCCAAACCAACCGGGGTTATCAGAACCTAAT
14521  GTGCTTAGCGTTAGTTAAAGACCAACCAGATTTGCAATTAGAACCCTTTTTGGATGGTAA
14581  TGTGGTAATTATTTGCACACAAACTGAACTGCGCCTCAACACCGCAAACCCCGTTTACTT
14641  AGCACATGGTTTGAGTGGTCGTTATCCTAAAATTGCAGTAACACAAAAACCAGTTAAGTG
14701  TCAAAACACCAATAAGGACTTAACACTGCTCTTAACACTTAAGCAAATTAGTCAAATTAA
14761  CAGCGAACACTTCCAGCCACTGGAATGAAAATTAAGCCGTAGTTTAAACGAAATCCAATT
14821  GGATCCACCATTGTTGCAACAACTCCGGCACCAACCTTTTTTAAGCCAAAAGGCAGCACA
14881  GCAAATTTTTAGCGAAGAGGAATTGGGAAATCTGCAAAAGCTAGTGGAGCAAAGTCAATG
14941  AGATTTAACC
```

1…363 (363 bp)
MPN368, partial
hypothetical protein

495…1256 (762 bp)
MPN369
conserved hypothetical protein

1897…4110 (2214 bp)
MPN370
hypothetical protein

4153…4788 (636 bp)
MPN371
hypothetical protein

4942…6717(1776 bp)
MPN372
similarity to pertussin toxin subunit S1

6728…7342 (615 bp)
MPN373
hypothetical protein

8302…9060 (759 bp)
MPN374
hypothetical protein

9406…9795 (390 bp)
MPN375

hypothetical protein

10175…13597 (3423 bp)
MPN376
hypothetical protein

14024…14248 (225 bp)
MPN377
hypothetical protein

14252…14950 (699 bp)
MPN378, partial
DNA polymerase III alpha subunit
dnaE

Table S1: Primers used for transcriptional analysis of *cards*

Primer name	Primer sequence	Note	
	**Primer extension**		
MPN372PE	GCTGTGGGTGTTTCTGAAGTGGAA	444484-444507[1]	144-167[2]
MPN372PE2	TCTCACATCACCTAAAGTTGA	444413-444433	73-93
MPN371PE	GCAAGTCCAACTCAAGGCGGACGAT	444038-444062	126-150
MPN373PE	CCGTAGTGCTTTGGTTTGGGTTGA	446639-446662	80-103
	**Reverse transcription PCR**		
MPN371P	GTGCCATCTTGGGTGCCTGA	444090-444109	337 bp (79-415)[3]
MPN371M	GCAATGTTTGGCTTGAAGG	443773-443791	
MPN372P[4]	TTCCACTTCAGAAACACCCACAGC	444484-444507	556 bp (144-699)
MPN372M[4]	TCAATCAGGGCACGCAAACG	445020-445039	
MPN373P	TCAACCCAAACCAAAGCACTACGG	446639-446662	401 bp (80-480)
MPN373M	ACCAGCTACCAACGGATCTACGTGG	446262-446286	
MPN393P[4]	ACTGGTTCTGCCCTACCTTCCGTTCC	471171-471196	456 bp (260-715)
MPN393M[4]	CTTCGTGCATTGCTTCGTAACTCGC	470741-470765	

[1] primer position within *M. pneumoniae* reference strain M129 genome
[2] primer position within corresponding gene
[3] size of the amplified fragment and its position within coding region
[4] primers used in quantitative real time RT-PCR

Chromosomal region of clinical isolate S1 used in this study, corresponding to nucleotides 439400 to 454348 of strain M129 complete genome was sequenced. The region contains identical coding regions, and only minor sequence differences were found. Sequence is provided in Fig. S1.

Table S2: Primers used for generating gene regions used in macroarray

Gene	Primer name	Primer sequence	Position within M129 genome	Amplicon (bp)	T_a[1] (°C)
dnaJ1	MPN002P	AGCGGAGTATGACGCTATGCTGCGC	2008-2032	496[2]	60
	MPN002M	TGTTGAGTTCGTACCACATTTGTCCAGGG	2476-2504	(171-667)	
dnaJ2	MPN021P	TTGCCCTATGATTTAGAGATTGCG	26553-26576	477	55
	MPN021M	ATTGGCTACTACATTATGGTCAACC	27005-27029	(394-870)	
dnaJ3	MPN119P	AATAAGGCACCGGATGCGGC	152682-152701	590	57
	MPN119M	TAACCGTACTCAGGATTACCAATCATTGC	153243-153271	(115-704)	
grpE	MPN120P	TTACCGAAATTCTCAGTTCCATCCG	155345-155369	554	55
	MPN120M	CGATACCTTCACCACCGTATTAGCCG	155873-155898	(23-576)	
hrcA	MPN124P	ACCCGACAAGCCCAAATTCTCAAGG	161746-161770	537	55
	MPN124M	GCGATCATTAAAGATCCTGACACATACC	161234-161261	(16-552)	
tig	MPN331P	TTGATGCCATCTTCCAGCCG	389146-389165	540	55
	MPN331M	TGATCTCTAACTGCTTAATTGCTTTGAGC	389657-389685	(224-763)	
lon	MPN332P	AGCGTTAGATAAGTTGTTGGAACGG	390705-390729	496	55
	MPN332M	TTTAATGTGCTCTGGATATGGG	391179-391200	(378-873)	
sigA	MPN352P	AAGGAGAGTGATGTACCAAAGAAGCGTCG	420002-420030	456	57
	MPN352M	GGAAGAATCGAGCGAACCTAGAAAGAAACG	419575-419604	(211-666)	
cards	MPN372P	TTCCACTTCAGAAACACCCACAGC	444484-444507	556	60
	MPN372M	TCAATCAGGGCACGCAAACG	445020-445039	(144-699)	
pdhB	MPN392P	GCTATGGCTGGTATTGGTGTGGGTGC	470140-470165	608	60
	MPN392M	GCCGCTTCGGTAACTACGAGCAAGC	469558-469582	(193-800)	
pdhA	MPN393P	ACTGGTTCTGCCCTACCTTCCGTTCC	471171-471196	456	57
	MPN393M	CTTCGTGCATTGCTTCGTAACTCGC	470741-470765	(260-715)	
nox	MPN394P	TTTGGGTTGTGGAATTGCCTTGG	472774-472796	557	57
	MPN394M	GGTTAATGCCCGCTTTCTTCATGG	472240-472263	(120-676)	
gap	MPN430P	TTAGCACACTTACTAAAGTACGACTCCGC	518687-518715	576	57
	MPN430M	AAGGGCCATACCGTTGAGCTTACC	518140-518163	(136-711)	
dnaK	MPN434P	CTACCGCCGCTGCATTGGCTTATGG	523137-523161	447	60
	MPN434M	AAACATCCGAAATTGGGTTGCGAGTACG	522715-522742	(464-910)	
clpB	MPN531P	AATTTATGAACTTTCGTTGTCTGG	653657-653680	427	55
	MPN531M	CATTTCTACAGCAGCTACCAAGGC	653254-653277	(270-696)	
groEL	MPN573P	GCTGTATCTACCAATGATATTGCTGG	697070-697095	493	55
	MPN573M	TTTCCATGCTACCTTCTAGTAACGG	696603-696627	(229-721)	
groES	MPN574P	AGTGCCCACAAGGGAACTGTCG	697541-697562	102	55
	MPN574M	AAAGTAGATCGTGTCACCGATGCC	697461-697484	(115-216)	
eno	MPN606P	AACTACGCGATGGTGATCCC	727353-727372	556	55
	MPN606M	GCTGCCTTAATTGCTTCGACC	726817-726837	(173-728)	
tuf	MPN665P	TCGTTGGTTTACGTCCAATC	788573-788592	130	55
	MPN665M	TCCACTTCTTTACGGTCCACA	788463-788483	(734-863)	
ftsH	MPN671P	GCCTACTACCAATTATTATCTTTGTCA	795873-795899	523	55
	MPN671M	TTTAAGGTTTGTTCGACCACCG	795377-795398	(533-1055)	
recA	MPN490P	TCGGCACTGGTGGTTTGCCACTAGG	597651-597675	517	60
	MPN490M	ATCCGCATCGAAGCGTAGAACTTCAACG	597159-597186	(152-668)	
P1	MPN141P	TATGATCCCACCCGAACCGAAGC	181188-181210	653	60
	MPN141M	TTGAGTTGGACGGACTGACCCG	181819-181840	(331-983)	
orf6	MPN142P	TCCGGGTCTAACTCCCAAGACTTCG	186122-186146	606	60
	MPN142M	ACTGACAGGTGCTGATCTCAAGAGCG	186702-186727	(376-981)	
P30	MPN453P	GCAGAAGAGGATACAGTACAAATTCAGGG	553325-553353	622	60
	MPN453M	CAGGACGTGGTGGTTGCATTCC	552732-552753	(166-787)	

[1] annealing temperature used in PCR.
[2] size of the amplified fragment and its position within the coding region

Fig. S2

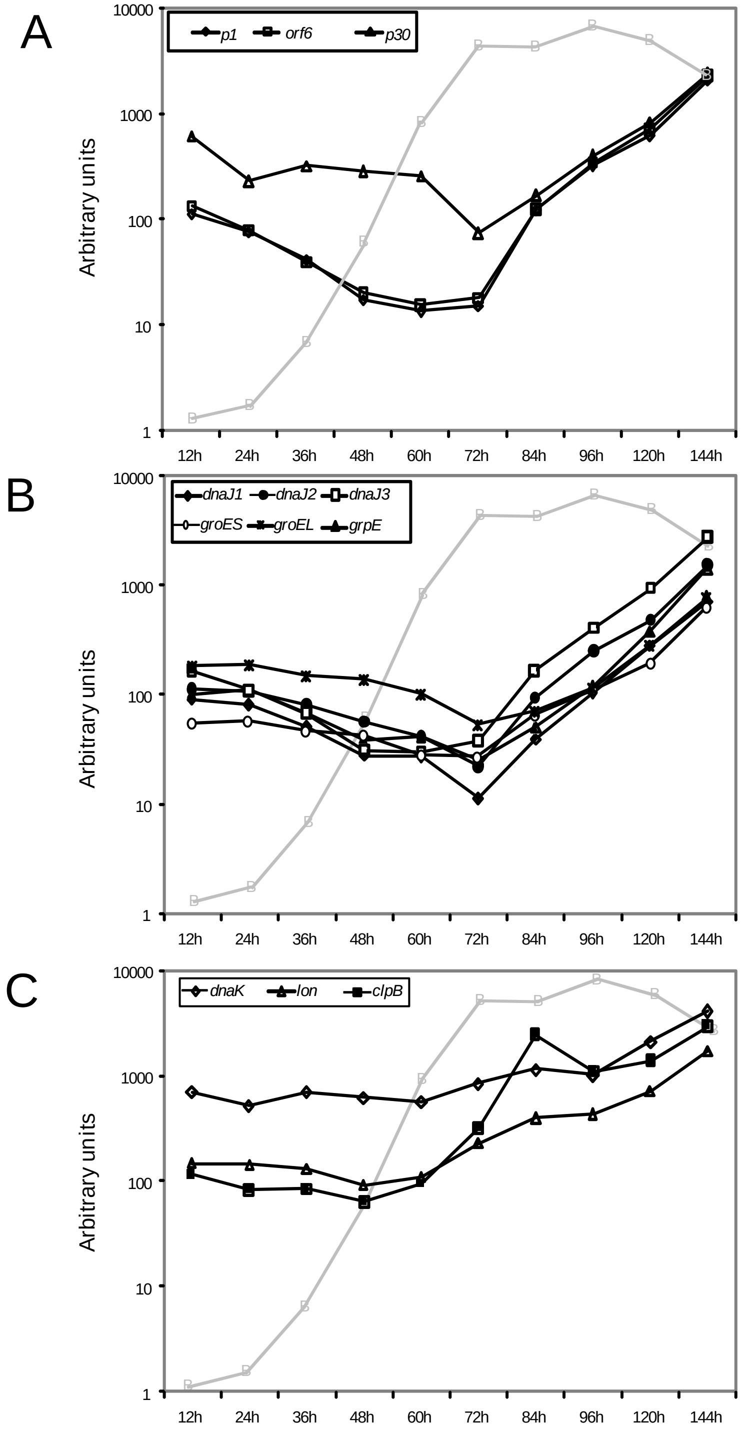

Growth phase-dependent variations in adherence (A) and heat-shock (B &C) related genes mRNA in *M. pneumoniae* SP-4 broth cultures. *M. pneumoniae* cells were grown at 37°C for 12, 24, 36, 48, 60, 72, 84, 96 and 120 h and harvested at each time interval for analysis. Experiments were repeated two times. Levels of each transcript are estimated by DNA slot blot (average value ± SD), and mycoplasma growth (total protein) are indicated in grey lines, and expression levels are presented in arbitrary values.
